# A narrative systematic review of definitions and diagnostic criteria for disordered eating and eating disorders in type 1 diabetes

**DOI:** 10.1007/s00125-026-06805-3

**Published:** 2026-07-29

**Authors:** Emmanouela Konstantara, Pil Lindgreen, Lisa Kuriakose, Natalie Zaremba, Rebecca Upsher, Janet Treasure, Khalida Ismail, Marietta Stadler

**Affiliations:** 1 Department of Diabetes, Faculty of Life Sciences and Medicine, King’s College London, London, UK; 2 Department of Prevention, Health Promotion and Community Care, Steno Diabetes Center Copenhagen, Herlev, Denmark; 3 Department of Psychological Medicine, Diabetes, Psychology and Psychiatry Research Group, King’s College London, London, UK; 4 Department of Medical Psychology, Radboud University Medical Center, Nijmegen, the Netherlands; 5 Department of Psychology, Institute of Psychiatry, Psychology and Neuroscience, King’s College London, London, UK

**Keywords:** Chronic illness, Diabulimia, Disordered eating, Feeding and eating disorders, Mental illness, Systematic review, Type 1 diabetes

## Abstract

**Aims/hypothesis:**

Type 1 diabetes and disordered eating (T1DE) affects 8–37.1% of adults and is associated with high rates of morbidity and mortality. The absence of a standardised case definition of T1DE and its severity hinders effective screening, diagnosis and treatment. This systematic review aimed to (1) synthesise existing case definitions and diagnostic criteria for T1DE in adults and (2) identify key characteristics to inform consensus for future diagnostic criteria.

**Methods:**

A systematic review was conducted following the Preferred Reporting Items for Systematic reviews and Meta-Analysis (PRISMA) guidelines. Eligible studies involved adults (≥18 years) with type 1 diabetes assessing disordered eating; paediatric studies, mixed samples without disaggregated data, non-empirical designs and non-English publications were excluded. PubMed, MEDLINE, EMBASE, CINAHL and PsycINFO were searched up to November 2025 for peer-reviewed studies involving adults with T1DE. Qualitative and quantitative data on definitions, diagnostic criteria and assessment tools were extracted. Study quality was appraised using a modified Graphical Appraisal Tool for Epidemiological studies (GATE) checklist. Due to heterogeneity of data, a narrative synthesis of findings was performed to describe current definitions of T1DE.

**Results:**

Sixty-one studies met the inclusion criteria, with a pooled sample of 111,208 participants (76% women) from over 22 countries. T1DE was defined using a heterogeneous array of terms, diagnostic frameworks and assessment tools (29 distinct methods). The Diabetes Eating Problem Survey-Revised (DEPS-R) was the most used questionnaire, but many studies relied on criteria adapted from general eating disorder classifications or generic questionnaires. Approximately three-quarters of the studies assessed insulin omission behaviours, but the operationalisation of the cognitions for insulin omission varied widely. Beyond physiological markers such as HbA_1c_ and BMI, studies explored various diabetes-related and psychological constructs, although often considering diabetes and disordered eating separately rather than as an integrated condition.

**Conclusions/interpretation:**

This systematic review highlights the lack of a unified, evidence-based definition of T1DE, resulting in inconsistent screening, diagnostic and reporting practices. Establishing clear, consistent, evidence-based diagnostic criteria and screening questionnaires for T1DE is critical to improving early detection and developing targeted interventions. These findings provide a foundation for refining T1DE definitions as a stepping stone to an international consensus definition.

**Study registration:**

PROSPERO registration no. CRD420250223622

**Funding:**

King’s College London and King’s College Hospital through the KMRT KCH Joint Research Committee studentship. This work was also conducted as part of the National Institute for Health Research (NIHR; CS-2017-17-023)-funded STEADY project (Safe management of people with Type 1 diabetes and EAting Disorders studY). NZ's salary was part-funded by the NIHR via the NIHR Clinician Scientist award to MS; JT and KI are part-funded by the NIHR Mental Health Biomedical Research Centre at South London and Maudsley NHS Foundation Trust and King's College London. MS was funded through her NIHR Clinician Scientist Fellowship (CS-2017-17-023).

**Graphical Abstract:**

**Figure:**
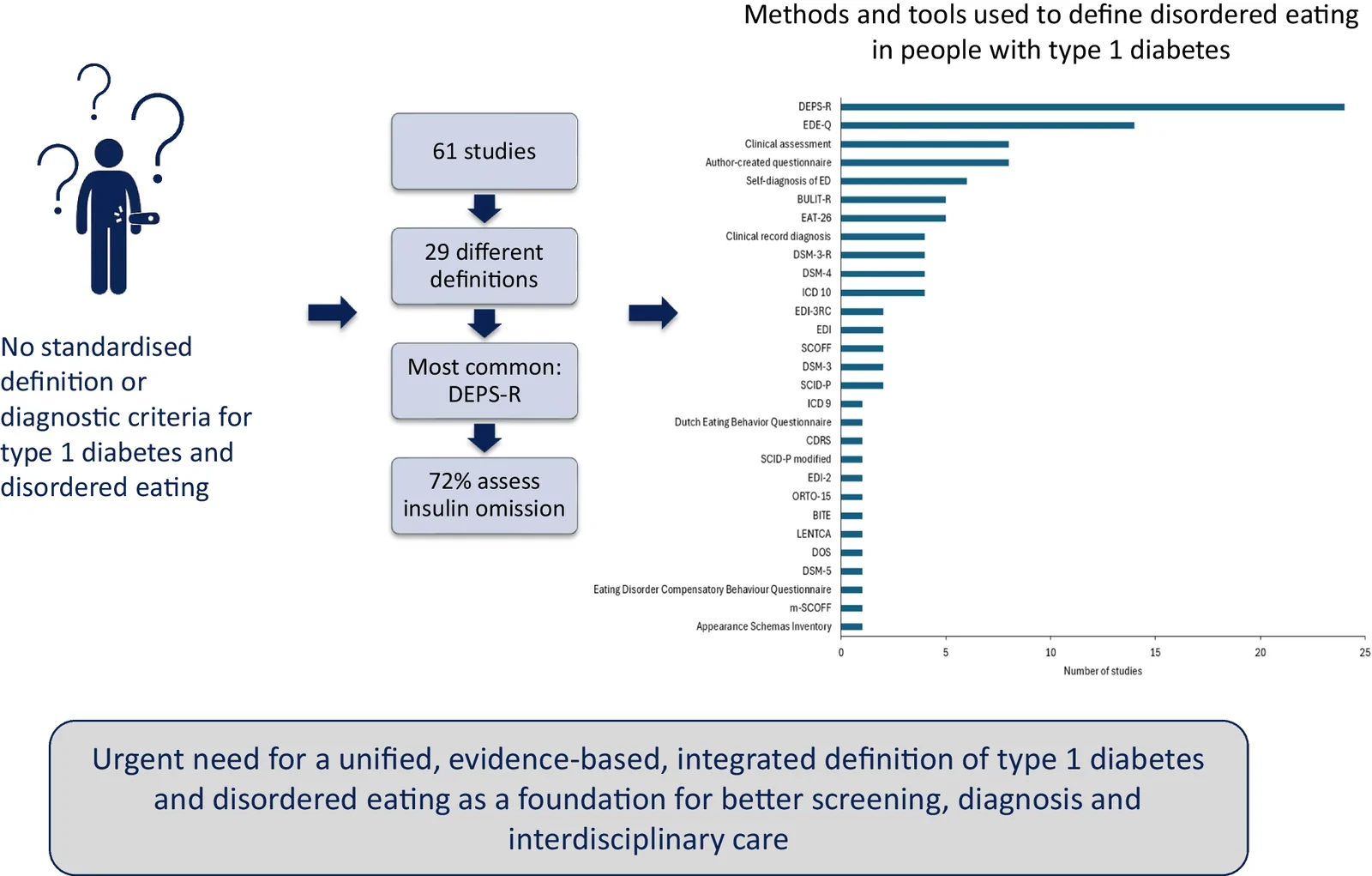

**Supplementary Information:**

The online version contains peer-reviewed but unedited supplementary material available at https://doi.org/10.1007/s00125-026-06805-3.

**Figure:**
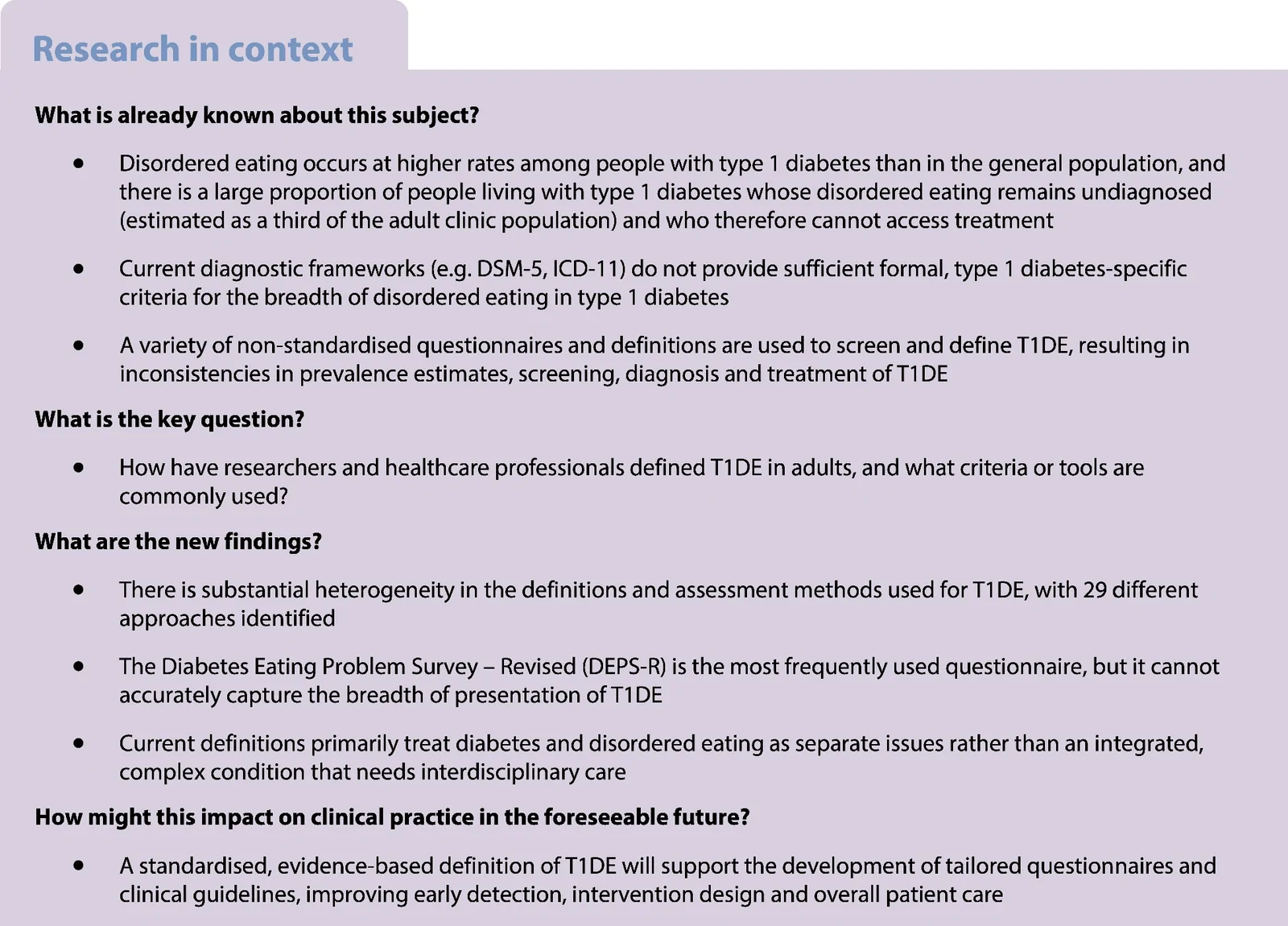

## Introduction

Type 1 diabetes is a lifelong autoimmune condition characterised by insulin deficiency and managed through insulin replacement therapy [1]. Self-management tasks, including carbohydrate counting, insulin dosing, frequent blood glucose monitoring and regular weighing, can heighten vulnerability to disordered eating behaviours by increasing the focus on food, weight and body shape [2]. This may be exacerbated by early post-diagnosis weight gain and the perception of insulin as a means of weight management [3, 4]. This is referred to as type 1 diabetes and disordered eating (T1DE) and encompasses behaviours such as intentional insulin underdosing or complete insulin omission for weight management, rigid dietary restriction, and binge eating related to hypoglycaemia management [5–7]. T1DE is associated with an increased incidence of diabetes-related complications [8, 9] and a threefold higher risk of mortality compared with type 1 diabetes alone [10, 11]. Despite its clinical significance, T1DE remains poorly defined, with no universally accepted diagnostic criteria [8, 12]. In this review, T1DE is used as an umbrella term for disordered eating behaviours and clinically diagnosed eating disorders in people with type 1 diabetes.

Existing diagnostic frameworks such as the Diagnostic and Statistical Manual of Mental Disorders, 5th edition (DSM-5), and the ICD-11 provide limited guidance on T1DE [12]. Early research in this field focused largely on adolescent populations [13] and relied on eating disorder questionnaires validated in people without diabetes [14], assessing metabolic and psychological outcomes separately without capturing the interplay between disordered eating behaviours, insulin use and glucose levels [15, 16]. Although recent studies conceptualise T1DE as a multifaceted condition [5, 9, 17], systematic reviews have primarily examined prevalence, risk factors or interventions [18], but have not synthesised how T1DE is defined, operationalised, and diagnosed in adults [8].

This gap is particularly important given that adults with type 1 diabetes face distinct diagnostic challenges, a longer disease duration, cumulative complications and different care pathways, all of which influence detection of T1DE [19]. The absence of a consistent definition hinders accurate prevalence estimates, delays detection, limits comparisons across studies and impedes the development of tailored treatments [12, 16].

This narrative systematic review aimed to identify and synthesise the range of criteria and screening practices used to define T1DE in adults. This will support the development of a more consistent, clinically relevant and internationally applicable consensus definition of T1DE.

## Methods

This narrative systematic review followed the Preferred Reporting Items for Systematic reviews and Meta-Analysis (PRISMA) guidelines [20] (PROSPERO registration no. CRD420250223622) (see electronic supplementary material [ESM] Table 1).

### Eligibility criteria

This review employed the PECOS framework (Population, Exposure, Comparator, Outcomes, Study design) [21–23] rather than the PICOS framework (Population, Intervention, Comparator, Outcomes, Study design), as participants are not intentionally ‘exposed’ to type 1 diabetes or disordered eating (Table 1). Studies were eligible for inclusion if they (1) involved adults (≥18 years) with type 1 diabetes; (2) assessed disordered eating behaviours, eating disorders or diabetes-specific eating-related behaviours (including insulin restriction or omission); and (3) reported original empirical data from peer-reviewed studies using quantitative, qualitative or mixed-methods designs. Studies were excluded if they (1) focused exclusively on children or adolescents; (2) included mixed populations without reporting separate data for people with type 1 diabetes; (3) did not assess disordered eating or eating disorder-related outcomes; (4) were non-empirical publications (e.g. reviews, editorials); or (5) were not published in English.

**Table 1:** Eligibility criteria according to the PECOS framework

PECOS framework	Inclusion criteria	Exclusion criteria
Population	• Adults with type 1 diabetes (≥18 years of age) • Healthcare professionals working with adults with type 1 diabetes	• People aged <18 years • People without type 1 diabetes • Healthcare professionals who do not work with adults with type 1 diabetes
Exposure	• Studies defining, characterising or screening for T1DE, including those focused on T1DE; comparing a T1DE population with other populations (e.g. type 1 diabetes without disordered eating); and exploring healthcare professionals’ perspectives	• Studies not focusing on T1DE • Studies focusing on conditions that could potentially be confounding (e.g. pregnancy, coeliac disease)
Comparator/comparison	• None	• None
Outcomes	• Mapped criteria to identify T1DE including physical (e.g. HbA_1c_, BMI), psychological (e.g. purging, eating disorder psychopathology) and other (e.g. socioeconomic status) criteria	• None
Study design	• Peer-reviewed, primary research studies with full-text availability in English • Cross-sectional, cohort, case–control, qualitative, mixed methods, ecological momentary assessment and experimental studies • Published from database inception to November 2025	• Grey literature • Opinion pieces • Posters, abstracts, conference papers • Studies without free full-text access • Case studies • Reviews • Published after November 2025

### Information sources and search strategy

PubMed, MEDLINE, EMBASE, CINAHL and PsycINFO were searched in September 2021, with updates in June 2023 and November 2025. Search terms identified studies referencing type 1 diabetes, disordered eating, insulin omission and diabulimia. Database-specific strategies using Medical Subject Headings (MeSH) were applied (ESM Table 2).

### Selection process

References were deduplicated in EndNote (version X9) [24]. Titles and abstracts were screened independently by EK, LK, NZ and PL, with discrepancies resolved by consensus with MS. Full texts were assessed for eligibility.

### Data collection process and data items

Data were extracted using an Excel form, including study aims, design, population characteristics, disordered eating, definitions and rationale for insulin omission, and physical and psychological outcomes. Extraction accuracy was reviewed by EK, LK and PL.

### Study risk of bias assessment

Study quality and risk of bias were assessed using the modified Graphical Appraisal Tool for Epidemiological studies (GATE) [25] as recommended by NICE [26]. GATE provides study design-specific checklists (e.g. for quantitative intervention studies, for quantitative studies reporting correlations); the most appropriate checklist was selected for each study. Appraisal comprised two levels of assessment. First, individual checklist items related to study design, conduct and reporting were rated as positive (++), unclear (+), negative (–), not reported (NR), or not applicable (NA). These item-level ratings were then used to determine overall study ratings for internal and external validity, reported as high (++), unclear (+) or low (–) (ESM Table 3).

### Synthesis methods

A narrative synthesis was conducted [27]. Data were grouped into conceptual domains and summarised using a frequency-based synthesis (vote counting). Quantitative findings were mapped to predefined domains to enable consistent aggregation and identify dominant and under-represented concepts relevant to T1DE definitions. Domains were refined through team discussion.

## Results

### Study selection

**Fig. 1:**
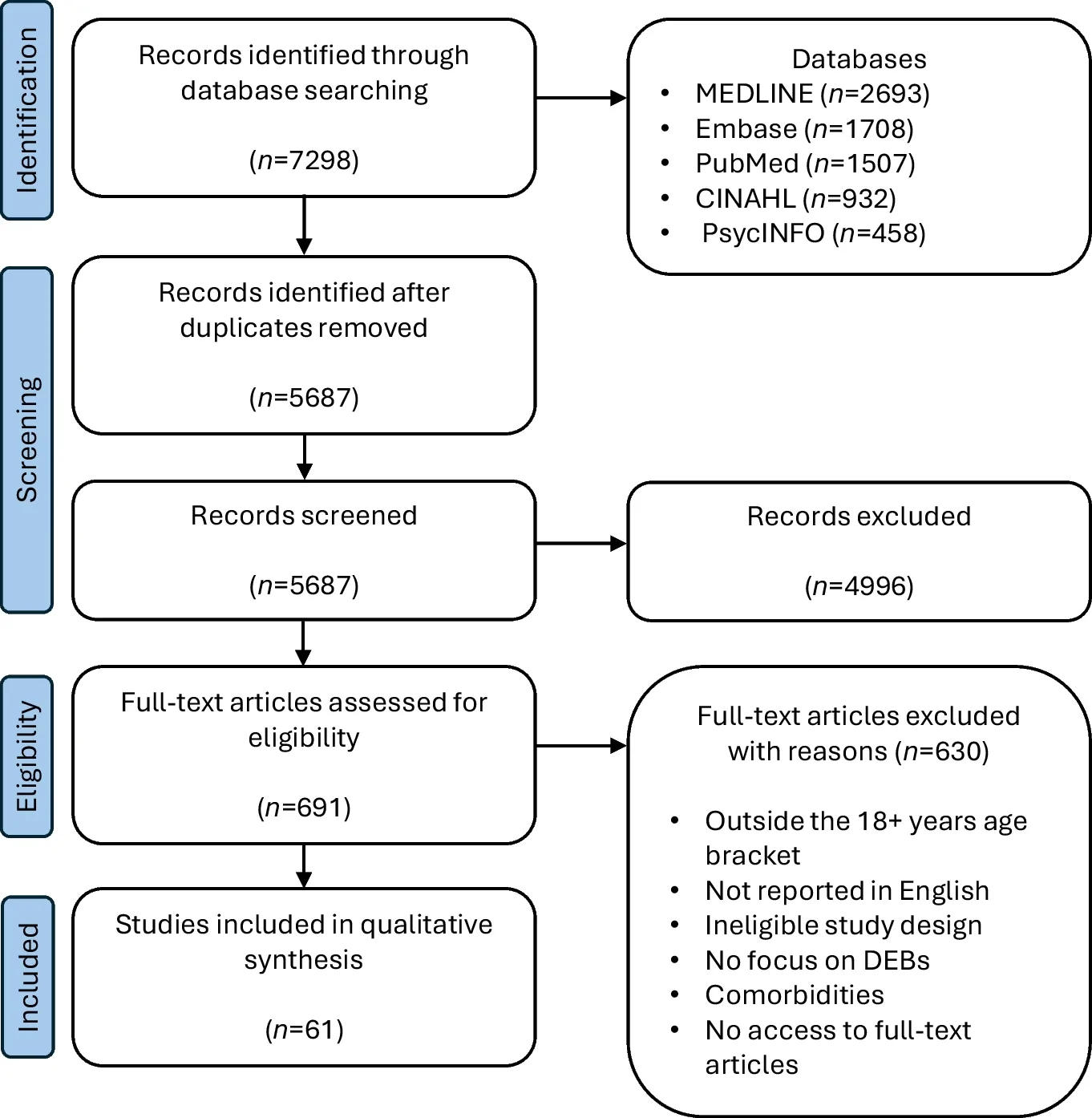
Flowchart of included studies. DEBs, disordered eating behaviours

A total of 7298 records were identified through electronic database searches (Fig. 1). After removing 1611 duplicates, 5687 titles and abstracts were screened, of which 691 full-text articles underwent review. Of these, 630 did not meet eligibility criteria, leaving 61 studies included in the final synthesis.

### Population characteristics

The 61 studies included 111,208 participants across more than 22 countries (Table 2, ESM Table 3). Of these, 27,419 people had type 1 diabetes, 70,071 people had no diabetes diagnosis, 867 people had type 2 diabetes, and 11,910 people had T1DE. Additionally, 821 people lived with an eating disorder but no diabetes, 45 were medical outpatients and 75 were healthcare professionals.

**Table 2:** Summary of included studies

Author, year, country	Study design	ED definition	Rationale	Insulin omission definition	Insulin omission assessed
Affenito (1997) USA [31]	Cross-sectional	DSM-3-R criteria using EDE-Q and BULIT-R	EDE-Q is a standardised psychometric instrument validated for a population with diabetes. DSM-3-R and BULIT-R: rationale NR	Intentional reduction or omission of prescribed insulin to induce glycosuria for weight management	Yes
Affenito (1997) USA [32]	Cross-sectional	DSM-3-R criteria using EDE-Q and BULIT-R	See above	See above	Yes
Affenito (1998) USA [33]	Cross-sectional	DSM-3-R criteria using EDE-Q and BULIT-R	See above	See above	Yes
Albaladejo (2023) France [34]	Cross-sectional	SCOFF questionnaire and one question from m-SCOFF	Validated in France for adults. Included a question from m-SCOFF specifically for insulin misuse that has not been validated	m-SCOFF question: ‘Do you ever take less insulin than you should?’	Yes
Apergi (2023) Greece [54]	Cross-sectional	Diabulimia: the voluntary restriction of insulin to lose weight, as measured by DEPS-R score	DEPS-R is specifically designed for the assessment of diabulimia risk in adults with T1D	Voluntary restriction of insulin to lose weight	Yes
Babayeva (2025) Turkey [63]	Cross-sectional	DEPS-R	DEPS-R has been translated and validated among Turkish adults with T1D	NR	No
Bächle (2015) Germany [80]	Cross-sectional	SCOFF questionnaire	Validated questionnaire for EDs, not for diabetes	NR	No
Bikri (2021) Morocco [75]	Cross-sectional	EAT-26	Arabic language version of EAT-26 scale has been validated in numerous studies and found to be highly reliable. EAT-26 described as ‘the most widely used test of eating psychopathology’	NR	No
Broadley (2018) Australia [35]	Cross-sectional	EDE-Q, DEPS-R	EDE-Q has been validated in a large Australian community sample, implemented successfully in T1D and displays good psychometric properties. DEPS-R: rationale NR	NR	No
d’Emden (2017) Australia [36]	Cross-sectional	EDI-3RC; Eating Disorder Compensatory Behaviour Questionnaire	NR	The Eating Disorder Compensatory Behaviour Questionnaire assesses the presence and frequency of insulin misuse	Yes
Embaye (2023) The Netherlands [55]	Cross-sectional	DEPS-R	DEPS-R has been designed for and validated in people with diabetes	Underdosing or omission of insulin can be considered an inadequate but highly effective compensatory behaviour, adopted by 20–40% of people with T1D to manage weight	No
Ercolino (2025) USA [64]	Cross-sectional	DEPS-R	DEPS-R has been validated in older adults with T1D	DEPS-R question 13: skipping insulin doses after overeating	Yes
Gawlik (2016) Australia [37]	Cross-sectional	Questionnaire created by authors asking about insulin restriction being used as a weight management strategy; Appearance Schemas Inventory	NR	Participants were asked whether they had ever engaged in insulin restriction and the purpose of this practice (‘no’, ‘yes—not for weight control’, ‘yes—for weight control’)	Yes
Hartlaub (2025) 22 countries, predominantly USA and UK [52]	Cross-sectional	EDE-Q, DEPS-R, single-item questionnaire created by authors to assess insulin restriction	EDE-Q and DEPS-R are validated measures widely used in diabetes–ED research. Insulin restriction item aligns with precedent in prior studies	Insulin restriction for the purpose of managing weight	Yes
Huisman (2023) The Netherlands [81]	Cross-sectional	DEBQ to assess binge eating	Satisfactory validity and reliability of the DEBQ have been published	Insulin restriction to influence weight (loss) is a particular DEB in T1D	No
Ip (2023) USA [90]	Cross-sectional	Self-reported	(1) Single-item inquiries to determine if participants took less insulin than prescribed, (2) Question on whether participants took less insulin specifically for weight management purposes	Diabulimia: individuals who reported intentionally skipping or using less insulin than directed for weight loss (or to prevent weight gain) in the past 12 months	Yes
Krzyżowska (2023) Poland [78]	Cross-sectional	EAT-26	NR	NR	No
Merwin (2014) USA [38]	Cross-sectional	DEPS-R and questionnaire developed by authors: behavioural and emotional indicators of disinhibited eating when blood glucose is perceived to be low	DEPS-R: ‘There is not an established measure that specifically assesses weight-related insulin mismanagement, there is a scale that assesses diabetes-specific ED symptomatology, which includes relevant items that may function as indicators of this form of insulin omission’. Authors’ questionnaire: ‘There are no established self-report measures that query the experience of eating when BG [blood glucose] is thought to be dropping or low. Thus, we developed and administered four individual items’	Insulin omission for weight management purposes	Yes
Merwin (2024) USA [7]	Cross-sectional	DEPS-R	Tool has been validated	Captured via DEPS-R questions	Yes
Priesterroth (2025) Germany [65]	Cross-sectional	DEPS-R	Tool has been validated	Captured via DEPS-R questions	Yes
Roberts (2025) USA [50]	Cross-sectional	DEPS-R, EDE 16.0	Tools have been validated	Insulin restriction for weight management	No
Stancin (1989) USA [39]	Cross-sectional	EDI and bulimia screening form comprising the DSM-3 bulimia diagnostic criteria	NR	NR	No
Tomás (2025) Portugal [77]	Cross-sectional	EAT-26	Tool has been validated	Omission of insulin for the purpose of weight loss	Yes
Usta Sağlam (2025) Turkey [49]	Cross-sectional	DEPS-R, EDE-Q	DEPS-R has been translated and validated in a Turkish sample. EDE-Q has been validated in a Turkish population of non-clinical adolescents	DEPS-R questions 4 and 13: skipping insulin doses after overeating	Yes
Watt (2022) Australia [67]	Cross-sectional	DEPS-R	DEPS-R has been developed specifically to assess DEBs in the diabetes population and includes factors such as insulin omission, so it was hypothesised that DEPS-R would be appropriate for use in the adult diabetes population	A common behaviour exhibited in people with T1D and DEB is insulin manipulation to induce hyperglycaemia and glycosuria, resulting in weight loss	Yes
Wisting (2018) Norway [56]	Cross-sectional	DEPS-R	DEPS-R has been translated and validated in Norwegian adolescents and adults with T1D	Insulin omission as a unique, T1D-specific compensatory behaviour. Potential risk factor for developing disordered eating	Yes
Wisting (2020) Norway [57]	Cross-sectional	DEPS-R	See above	Intentional insulin omission for weight management	Yes
Wisting (2021) Norway [58]	Cross-sectional	DEPS-R	See above	Intentional insulin omission for weight management	Yes
Colton (2015) Canada [40]	Cohort	EDE-Q, DSM-4 criteria, EDNOS diagnostic criteria for those with T1D	NR	Insulin omission to lose weight	Yes
Dickens (2015) USA [74]	Cohort	EDI-3	EDI-3 has been demonstrated to have high reliability and good construct validity	Purging through insulin omission for weight loss purposes	No
Doyle (2017) USA [59]	Cohort	DEPS-R	DEPS-R has good reliability and validity	Insulin omission for weight management is a DEB	Yes
Erthal (2022) Brazil [76]	Cohort	EAT-26	EAT-26 has been translated and validated in the Portuguese population	NR	No
Goebel-Fabbri (2008) USA [10]	Cohort	BULIT-R, EDI	Both measures used in previous diabetes research	Self-reported insulin restriction status	Yes
Goebel-Fabbri (2011) USA [41]	Cohort	BULIT-R and author-devised self-report measure for diabetes-specific eating and weight concerns	Author used revised measures due to social desirability pressures that could influence women to under-report insulin restriction as a symptom, particularly as part of research performed in a specialty diabetes centre	Intentionally taking less insulin than prescribed aimed at calorie purging and weight loss. Responses to the screening statement, ‘I take less insulin than I should’, to determine insulin restriction status	Yes
Hirvelä (2025) Finland [86]	Cohort	ICD-10	Standard ICD-10 codes captured in the national health insurance database	NR	No
Ismail (2024) UK [9]	Cohort	Definition developed by authors: ‘Severe T1D and disordered eating as a diagnosis of T1D, pervasive fear of insulin as a cause of weight gain, omission of insulin to manage weight, and at least one severity indicator of either a HbA_1c_ concentration of 10% or higher (≥86 mmol/mol) in the past 12 months, recurrent DKA (more than one in the past 2 years), or recurrent SH, or BMI of 15 kg/m^2^ or lower in the past 12 months’	Existing DSM-5 and ICD-11 categories (anorexia/bulimia) do not incorporate diabetes-specific behaviours, especially insulin omission. The team synthesised data from qualitative studies, patient narratives, interviews and clinical observations to build a T1D-specific psychopathology model	Insulin omission to manage weight	Yes
Kim (2024) Republic of Korea [85]	Cohort	ICD-10	Standard ICD-10 codes captured in the national health insurance database	NR	No
Takii (2003) Japan [84]	Cohort	SCID-P modified to follow DSM-IV 4 criteria	NR	Insulin omission as an ‘inappropriate compensatory behaviour to prevent weight gain’, defined as omission of more than one in four injections or the whole prescribed dosage of insulin to prevent weight gain	Yes
Takii (2008) Japan [83]	Cohort	SCID-P	NR	As above	Yes
Cooper (2017) Australia [42]	Case–control	ICD-9 and ICD-10. informal clinical interviews, based on codes	Method used to code mental health diagnoses in the databases used	NR	No
Cosentino (2023) Italy [43]	Case–control	ORTO-15, DOS, EDE-Q	NR	NR	No
Custal (2014) Spain [44]	Case–control	Clinical interview using DSM-IV under the EDNOS category: (1) participants with sub-threshold anorexia nervosa (who fulfil the diagnosis of anorexia), (2) those with sub-threshold bulimia nervosa (who fulfil the diagnosis of bulimia), (3) those with ‘pure’ EDNOS and psychological tests including EDI-2	NR	Insulin misuse was identified if participants admitted to manipulating or omitting their insulin dose as a purging method	Yes
Friedman (1998) France [45]	Case–control	EAT; BITE based on DSM-3 criteria for bulimia; clinician-based diagnostic evaluation using LENTCA, which bases diagnosis on DSM-3-R	NR	Lowering insulin dosage to prevent weight gain	Yes
Keane (2018) Ireland [71]	Case–control	EDE-Q	NR	Insulin misuse for weight management	No
Shah (2018) USA [87]	Case–control	Clinic-reported ED	NR	Participant-reported data to assess compliance with prescribed medical regimens based on the frequency of missed insulin doses (basal and bolus) and number of days with physical activity lasting >30 min	Yes
Ward (1995) UK [28]	Case–control	Previous or current diagnosis of ED as per clinical records	NR	Manipulating insulin to manage weight and shape	Yes
Balfe (2013) Ireland [29]	Qualitative	Reported previous ED diagnosis or considered themselves to have had ED previously	NR	Insulin omission to lose weight	Yes
Embick (2025) UK [82]	Qualitative	Self-reported: (1) fear or disturbance regarding one’s body weight or shape; (2) direct (taking less insulin than prescribed) or indirect restriction of insulin (reducing the need for insulin through dietary restriction); may include the use of compensatory behaviours such as self-induced vomiting, laxative use and excessive exercise to manage weight. (1) and (2) are causing harm to health, diabetes distress or impairments in functioning	Definition used in another project study (ComPASSION). Authors state this is not a final definition of T1DE; they used it because it is the first term that recognises and conceptualises the difficulties individuals face when living with both conditions	Direct (taking less insulin than prescribed) or indirect (reducing the need for insulin through dietary restriction) insulin restriction	Yes
Hage (2023) Norway [66]	Qualitative	DEPS-R	NR	Insulin omission assessed via DEPS-R	Yes
Harrison (2021) UK [46]	Qualitative	Clinical assessment with a consultant diabetologist and clinical psychologist; DEPS-R	NR	Broad definition of insulin omission behaviour and cognition, inclusive of direct and indirect insulin restriction, or omission; insulin restriction because of fear of weight gain or fear of hypoglycaemia	Yes
Harrison (2024) UK [4]	Qualitative	Self-reported; DEPS-R; clinical assessment with a consultant physician and a health psychologist, reviewed by a clinical psychologist with expertise in EDs	DEPS-R: >20 is the clinical cut-off; rationale for other methods NR	Insulin omission as a means of preventing weight gain or provoking weight loss	Yes
Hastings (2016) UK [88]	Qualitative	Participants self-categorising as recovering from diabulimia	NR	Deliberate and chronic withholding of insulin specifically and only for weight loss	Yes
Macdonald (2018) UK [30]	Qualitative	Current or previous diagnosis of ED as defined by DSM-5	NR	Omission/reduction of insulin to manage weight and compensate for eating	Yes
Falcão (2017) Portugal [47]	Mixed methods	EDE-Q; CDRS; questionnaire designed by the authors to evaluate, among other things, deliberate insulin omission (e.g. reasons, duration, reaction of others, awareness of the consequences and support received)	EDE-Q is available in Portuguese and has good internal consistency. CDRS is available in Portuguese and has good validity. Insulin omission questionnaire: rationale NR	Diabulimia: intentional omission or reduction of insulin to lose weight	Yes
Rama Chandran (2021) UK [89]	Mixed methods	Self-declared as having disordered eating	NR	Insulin restriction or omission of basal or bolus insulin to lose weight	Yes
Verbist (2021) UK [62]	Mixed methods	BISS, DEPS-R	Strong internal consistency and construct validity. DEPS-R has been empirically established as a threshold of systematic presentation of DEBs and insulin mismanagement. BISS: rationale NR	Recommended cut-off score of ≥20 on DEPS-R	Yes
Merwin (2015) USA [60]	EMA	DEPS-R score ≥20 initially, then also included DEPS-R score <20	DEPS-R is a diabetes-specific measure that assesses insulin restriction to manage weight. Included individuals with a range of DEPS-R scores to capture the continuum of ED pathology	Intentional insulin restriction as a maladaptive weight management strategy (purgative behaviour). Insulin restriction at each eating occasion was defined as responding ‘no’ to the question, ‘Did you take enough insulin to cover your food?’	Yes
Merwin (2018) USA [48]	EMA	DEPS-R score	DEPS-R is a diabetes-specific self-report measure of ED symptoms. Scores of ≥20 have been associated with higher HbA_1c_	Intentional insulin restriction as a maladaptive weight management strategy (purgative behaviour). Insulin restriction was defined based on the response to the question: ‘You said that you ate [breakfast] [X times]. How many times over the past four weeks did you provide yourself with sufficient/insufficient insulin to cover your eating for this meal?’ Omitting insulin due to hypoglycaemia and taking less insulin unintentionally (e.g. miscalculation of insulin needs) were not counted as episodes of insulin restriction. EDE was used as a secondary measure of reporting insulin restriction	Yes
Moskovich (2019) USA [61]	EMA	DEPS-R score ≥20 initially, then also included DEPS-R score <20	DEPS-R has demonstrated excellent internal consistency and external validity	Intentional restriction of insulin to induce glycosuria	Yes
Broadley (2019) Australia [70]	Quasi-experimental	EDE-Q	EDE-Q has been validated in non-clinical and T1D populations in Australia. Internal consistency for the EDE-Q Global scale in the present sample was high	NR	No
Stadler (2025) UK [51]	Feasibility RCT	Clinical interview using SCID-5RV to assess inclusion as per DSM-5 or ICD-11; additional T1DE-specific criteria developed by the study team; EDE-QS; DEPS-R	Criteria reflect validated ED screening tools (DEPS-R, EDE-QS) and clinical diagnostic standards (DSM-5/ICD-11)	Intentional insulin restriction or omission for weight management or avoidance of hyperglycaemia fluctuations	Yes

BISS, Body Image States Scale; BITE, Bulimic Investigatory Test of Edinburgh; BULIT-R, Bulimia Test-Revised; CDRS, Contour Drawing Rating Scale; DEB, disordered eating behaviour; DEBQ, Dutch Eating Disorder Questionnaire; DEPS-R, Diabetes Eating Problem Survey-Revised; DKA, Diabetic Ketoacidosis; DSM, Diagnostic and Statistical Manual of mental disorders; DOS, Düsseldorf Orthorexie Scale; EAT-26, Eating Attitudes 26; ED, eating disorder; EDE 16.0, Eating Disorder Examination Questionnaire 16; EDE-Q, Eating Disorder Examination Questionnaire; EDE-QS, Eating Disorders Examination Questionnaire-Short; EDI-3RC, Eating Disorder Inventory Risk Composite; EDNOS, Eating Disorder Not Otherwise Specified; EMA, ecological momentary assessment; ICD, International Classification of Diseases; LENTCA, Logistic System for Nosographic Evaluation of Eating Disorders; m-SCOFF, modified Sick, Control, One stone, Fat, Food; NR, not reported; ORTO-15, Ortorexie-15; SCID-5RV, Structured Clinical Interview for DSM 5 – Research Version; SCID-P, Structured Clinical Interview for DSM disorders; SCOFF, Sick, Control, One stone, Fat, and Food; SH, severe hypoglycaemia; T1D, type 1 diabetes

Gender/sex data were reported in 60 studies; 18 included only female participants. In total, 76% of participants were women. One study [28] did not report gender/sex, and two studies [29, 30] reported gender/sex for participants with type 1 diabetes but not for healthcare professionals (ESM Table 3).

### Study characteristics

Of the 61 studies, 59 were observational and two were experimental. Observational designs included 28 cross-sectional, 11 cohort and seven case–control studies, as well as seven qualitative, three mixed-methods and three ecological momentary assessment (EMA) studies. One quasi-experimental study and one feasibility randomised controlled study were also included. One study spanned 22 countries, predominantly the USA and the UK. Eighteen studies were conducted in the USA, 11 in the UK, six in Australia, four in Norway, two each in France, the Netherlands, Ireland, Japan, Germany, Portugal and Turkey, and one each in Brazil, Canada, Greece, Italy, Morocco, Spain, Poland, Republic of Korea and Finland. Publication dates ranged from 1989 to 2025 (ESM Table 3).

### Quality appraisal

Overall study quality was good, with internal validity generally higher than external validity (ESM Table 3). Qualitative studies scored well; six had a low risk of bias for external validity and one was unclear. For the 54 quantitative studies, external validity varied: about 33% had a low risk of bias, 59% an unclear risk and 7% a high risk. Internal validity was generally stronger than external validity, with 48% showing a low risk of bias, 48% an unclear risk and 4% a high risk. Cross-sectional and cohort studies displayed a wide range of quality, with most meeting several validity criteria but some showing unclear risks due to limited representativeness or potential confounding. Case–control studies scored well, with five having good internal and external validity. Mixed-methods and EMA studies were generally well conducted, although applying appraisal checklists not designed specifically for mixed methods introduced interpretive bias.

### Definition and classification of T1DE

**Fig. 2:**
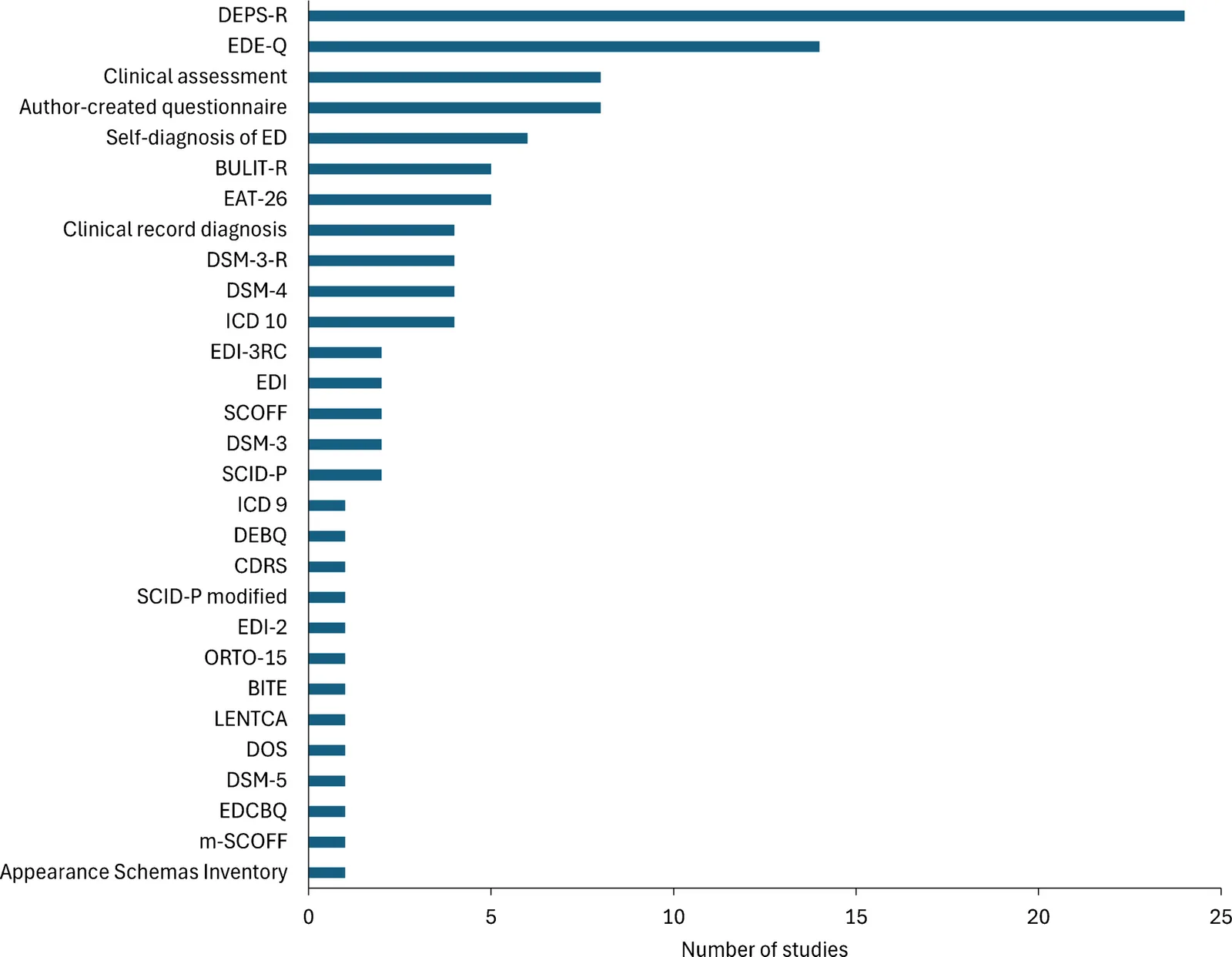
Methods and tools used to define disordered eating in people with type 1 diabetes. BITE, Bulimic Investigatory Test of Edinburgh; BULIT-R, Bulimia Test – Revised; CDRS, Contour Drawing Rating Scale; DEBQ, Dutch Eating Behavior Questionnaire; DEPS-R, Diabetes Eating Problem Survey – Revised; DOS, Düsseldorf Orthorexie Scale; EDCBQ, Eating Disorder Compensatory Behaviour Questionnaire; EDE-Q, Eating Disorders Examination Questionnaire; EDI, Eating Disorder Inventory; EAT-26, Eating Attitudes 26; ED, eating disorder; EDI-3RC, Eating Disorder Inventory Risk Composite; LENTCA, Logistic System for Nosographic Evaluation of Eating Disorders; m-SCOFF, modified Sick, Control, One stone, Fat, Food; ORTO-15, Ortorexie-15; SCID-P, Structured Clinical Interview for DSM Disorders; SCOFF, Sick, Control, One stone, Fat, and Food

In total, the 61 included studies used 29 different approaches to identify or define T1DE (Fig. 2). These approaches included validated and non-validated questionnaires, clinical assessments, diagnostic classification systems clinical records and patient self-diagnosis. Approximately 40% of the studies (*n*=24) applied multiple approaches [4, 10, 31–52] to supplement their primary screening measure for T1DE. For example, Harrison et al [46] combined a validated diabetes and disordered eating questionnaire with a clinical assessment.

While some studies employed diabetes-specific questionnaires, others adapted general eating disorder questionnaires that were not standardised for people with type 1 diabetes. Researchers sometimes combined a standardised questionnaire with clinical interviews or extracted diagnoses from medical records. In other studies, participants self-reported T1DE symptoms or identified themselves as having ‘diabulimia’, which is not formally recognised as a distinct diagnosis. The different classification approaches are described in the following sections.

#### Validated questionnaires

The Diabetes Eating Problem Survey – Revised (DEPS-R) [53] was the most frequently used questionnaire, used in 24 studies [4, 7, 35, 38, 46, 48–52, 54–67]. Authors reported that the DEPS-R had good reliability and validity and captured T1DE behaviours more accurately than general eating disorder questionnaires [68]. Some studies adopted the DEPS-R as their primary measure; others paired it with additional questionnaires or clinical assessment frameworks.

The Eating Disorder Examination Questionnaire (EDE-Q) [69] was used in 14 studies [31–33, 35, 40, 43, 47, 49–52, 60, 70, 71], sometimes alongside DSM or ICD criteria. The EDE-Q and its interview-based version, the EDE, were selected because they are validated for eating disorders in the general population and have good internal consistency in populations with type 1 diabetes [69]. When paired with clinician-based interviews, authors believed that the EDE-Q detected subclinical symptoms.

The Bulimia Test – Revised (BULIT-R) [72], a tool assessing bulimic symptomatology that is grounded in DSM-3-R criteria for bulimia nervosa, was used in five studies [10, 31–33, 41].

The Eating Disorder Inventory (EDI) [73] and its variations (EDI-3RC, EDI-2) were employed in five studies [10, 36, 39, 44, 74], with authors selecting them for their robust psychometric properties.

Other validated questionnaires adapted from the general eating disorder literature included the Eating Attitudes Test (EAT-26) [14], applied in five studies [54, 75–78], and the Sick, Control, One stone, Fat, Food (SCOFF) questionnaire [79], used in two studies [34, 80]. Five more validated questionnaires were used in one study each: the Bulimic Investigatory Test of Edinburgh (BITE) [45], the Appearance Schemas Inventory [37], the Dutch Eating Behaviour Questionnaire [81], the Düsseldorf Orthorexie Scale (DOS) [43], and the Contour Drawing Rating Scale (CDRS) [47].

#### Non-validated questionnaires

Non-validated questionnaires, each used in a single study, included a modified version of SCOFF (m-SCOFF) [34], Ortorexie-15 (ORTO-15) [43] and the Eating Disorder Compensatory Behaviour Questionnaire [36]. These were employed to capture aspects not fully represented in validated tools, such as body image distortions, orthorexia or compensatory behaviours such as insulin restriction.

#### Author-generated questionnaires

In eight studies, authors developed their own questionnaires to assess behaviours or cognitions specific to T1DE [9, 37, 38, 41, 44, 51, 63, 82]. Two of these [37, 44] focused on insulin restriction for weight management. Another two developed a severity-based diagnosis that incorporated psychological and biomedical criteria [9, 51]. One study [38] assessed disinhibited eating when participants perceived that their blood glucose was dropping, one study [41] asked participants to rate their insulin restriction frequency on a Likert scale, and another study developed criteria that assessed disturbance regarding body weight or shape and direct or indirect insulin restriction [82].

#### Diagnostic classification systems

Fourteen studies referenced formal psychiatric classification systems to define or categorise disordered eating: versions of the DSM were used in 11 studies [30–33, 39, 40, 44, 45, 51, 83, 84] and versions of the ICD were used in four studies [42, 51, 85, 86]. The DSM and ICD are considered gold standards for diagnosing mental disorders, but neither provides diabetes-specific criteria for disordered eating. Researchers therefore adapted these criteria, based on team consensus, focusing on behaviours such as insulin omission.

#### Clinical assessments

Clinical assessments were employed by eight studies, often with structured questionnaires [4, 42, 44–46, 51, 83, 84]. The structured interview, Structured Clinical Interview for DSM Disorders (SCID-P) [83], or a modified version thereof [84], was aligned with DSM-4 criteria. One study used the Logistic System for Nosographic Evaluation of Eating Disorders (LENTCA) [45], a diagnostic questionnaire based on DSM-3-R criteria.

#### Clinical records

Four studies [28, 30, 42, 87] collected information from clinical records, identifying T1DE via codes or physician-documented diagnoses. These records sometimes noted ‘diabulimia’ or unspecified eating disorders in patients with type 1 diabetes, but without the use of standardised criteria documentation practices were inconsistent.

#### Participant self-diagnosis

Six studies relied on participant self-diagnosis [29, 30, 88–90], allowing participants to declare they had disordered eating or diabulimia. While this approach provided insight into participants’ lived experiences, it lacked validation against objective criteria and might not have captured the full range of disordered behaviours.

### Definition and assessment of insulin omission

Insulin omission was specifically assessed in 44 (72%) studies. While all studies agreed that the term entailed that insulin was deliberately underdosed or withheld to influence weight, the operational definitions varied widely. Some studies applied strict thresholds, such as omitting more than one-quarter of prescribed injections to influence weight [83, 84]. Others used a Likert scale or a single yes/no question [10, 37, 41] to determine insulin restriction status. Some considered insulin omission intentional only if it was explicitly weight related, whereas others counted any insufficient dosing as insulin restriction.

Merwin et al changed their approach between studies [48, 60]. Initially, they asked if participants took ‘enough insulin to cover your food’. Later, they refined the question to assess insulin sufficiency for each meal over 4 weeks, excluding unintentional miscalculations or omissions due to hypoglycaemia. Harrison et al [46] distinguished between direct omission for weight management and indirect omission motivated by fear of hypoglycaemia, highlighting that not all insulin manipulation is solely about losing weight. Albaladejo et al [34] embedded insulin omission in a modified SCOFF question. Such variability in definitions and metrics made comparing rates of insulin omission across studies challenging.

### Key aspects of T1DE

The included studies examined outcomes relevant to understanding and identifying T1DE (Table 3). During data extraction, these outcomes were categorised as ‘diabetes related’ or ‘disordered eating related’, reflecting how studies often measured them separately despite their co-occurrence in T1DE. These outcomes were counted and grouped into conceptual domains within these two overarching categories.

**Table 3:** Key characteristics for the diagnosis of T1DE

Author, year, country	Study design	Aim	Diabetes-related outcomes/measures	Other outcomes/measures
Affenito (1997) USA [31]	Cross-sectional	To examine lipid parameters that are affected in women with IDDM who engaged in disordered eating behaviours	HbA_1c_, triacylglycerol, compliance to prescribed insulin regimen, BMI	Dietary intake (NCI FFQ); EDE-Q; BULIT-R; anthropometric data; biochemical tests
Affenito (1997) USA [32]	Cross-sectional	To characterise the relationship between subclinical and clinical EDs and HbA_1c_ in women with IDDM	HbA_1c_, BMI, diabetes duration, diabetes onset, prescribed number of insulin units, daily injections, daily testing, insulin misuse, diabetes self-care, SMBG, frequency of minor and major insulin reactions, diabetes hospital admissions, insulin administration, diabetes-related complications, compliance to prescribed insulin regimen	Exercise habits, dietary patterns, age, ethnicity, occupation, EDE-Q, BULIT-R
Affenito (1998) USA [33]	Cross-sectional	To explore the relationship between insulin misuse and EDs with regard to body weight, body composition, body fat distribution and other related variables	Attitudes and behaviours regarding insulin misuse	Attitudes and behaviours regarding body composition measurements; anthropometric measurements, biochemical assessment, EDE-Q, BULIT-R
Albaladejo (2023) France [34]	Cross-sectional	To determine the prevalence of likely EDs and insulin misuse in a prospective cohort of adults with T1D treated with insulin pump therapy	Insulin pump and CGM data (time in range, mean amplitude glycaemic excursion), insulin misuse (m-SCOFF), questions related to diabetes	Information about lifestyle, medical history, SCOFF, IPAQ, eating habits, anthropometric data
Apergi (2023) Greece [54]	Cross-sectional	To explore possible lifestyle and diet factors associated with diabulimia in individuals with T1D	DEPS-R, HbA_1c_, BMI, diabetes self-management (number of daily SMBG, CGM use, number of hypoglycaemic events per week, total insulin units per day), diabetes nutrition management, carbohydrate counting method, tools for carbohydrate counting	EAT-26, BDI-II, weight, height, demographic data, weight attitudes, exercise frequency, eating/skipping breakfast, frequency and size of meals/snacks, being under dietitian’s supervision/counselling, being on diet, medical history
Babayeva (2025) Turkey [63]	Cross-sectional	To investigate the prevalence of DEBs in individuals with T1D and T2D on intensive insulin therapy followed in an outpatient clinic and identify actions that increase the risk of possible DEBs in the population with T1D and T2D	BMI, HbA_1c_, diabetes duration, total daily insulin dose, use of CSII, DEPS-R, frequency of hypoglycaemia	Age, sex, weight
Bächle (2015) Germany [80]	Cross-sectional	To analyse the prevalence of EDs and their association with depressive symptoms in a well-defined group of young adults with early-onset, intensely treated T1D of long duration, and estimate the associations between symptoms of EDs, depressive symptoms and metabolic management	HbA_1c_, BMI	PHQ-9, SES, professional status, household income, information on family structure/residence, SCOFF
Bikri (2021) Morocco [75]	Cross-sectional	To determine whether sociodemographic and clinical characteristics, eating attitude and psychoemotional characteristics can predict visual perception and working memory impairment in a Moroccan population with T1D	Fasting glucose, glucose levels, HbA_1c_, biochemical analysis (triacylglycerol), cholesterol, BMI	Sociodemographic data, systolic and diastolic BP, height and weight, biochemical analysis (creatinine), eating attitudes (EAT-26), mental health symptoms (DASS-21); cognitive evaluation (ROCF-A to evaluate visual perception and working memory)
Broadley (2018) Australia [35]	Cross-sectional	To determine the association between executive function and disordered eating in young adults with T1D vs a control group without diabetes	DEPS-R	EDE-Q, BRIEF-A, FES, DASS-21
d’Emden (2017) Australia [36]	Cross-sectional	To profile demographic, medical and psychosocial characteristics of young people with diabetes and develop a screening tool and care pathway for routine use	Eating Disorder Compensatory Behaviour Questionnaire assessing purging behaviours including insulin misuse; PAID; HbA_1c_; insulin administration modality; diabetes service use	CD-2; MSPSS; PSS; Kessler 10 assessing psychological distress; quality of life; EDI-3RC; financial concerns
Embaye (2023) The Netherlands [55]	Cross-sectional	Examine the associations between DEBs, diabetes distress and emotion regulation strategies in the context of T1D	DEPS-R, PAID-5	ERQ
Ercolino (2025) USA [64]	Cross-sectional	To explore the prevalence of DEBs and attitudes among older adults with T1D and associations with demographic and clinical variables	DEPS-R, HbA_1c_, BMI, diabetes duration, insulin pump use, CGM use, hybrid closed-loop use	Demographics , living situation, caregiver involvement, employment/retirement status, nutrition counselling history
Gawlik (2016) Australia [37]	Cross-sectional	To evaluate associations between the appearance investment component of body image, age, quality of life and self-reported metabolic management, along with the practice of insulin restriction as a weight management strategy	Age at diabetes diagnosis, disease duration, Diabetes Quality of Life Brief Clinical Inventory, HbA_1c_, insulin misuse	Appearance Schemas Inventory, age
Hartlaub (2025) 22 countries, predominantly USA and UK [52]	Cross-sectional	To examine psychosocial correlates (diabetes distress, weight esteem, physical appearance comparisons on social media, perception of BMI as a health indicator) of dietary restraint, diabetes-specific disordered eating and intentional insulin restriction among young adults with T1D	EDE-Q, DEPS-R, intentional insulin restriction, demographic diabetes information (method of insulin delivery, age at diagnosis), DDS-17	Weight esteem (BESAA), physical appearance comparisons on social media (modified PACS-R), perception of BMI as a health indicator, sociodemographic information, ED diagnoses (previous diagnosis, management of condition), self-reported mental and physical health
Huisman (2023) The Netherlands [81]	Cross-sectional	To examine the prevalence and health risks of binge eating in people with diabetes	Type of diabetes, diabetes duration, diabetes treatment, HbA_1c_, hospitalisations, SH in past 12 months, severe hyperglycaemia in past 12 months, diabetes-related complications, PAID, BMI	Gender, age, weight and height, formal diagnosis of depression, anxiety or ED, use of related medication and psychological/psychiatric care, PHQ-9, GAD-7
Ip (2023) USA [90]	Cross-sectional	To determine the prevalence of diabulimia among adults with T1D, differences between adults with and without diabulimia and risk factors associated with diabulimia	HbA_1c_, diabetes-related emergency department visit or hospitalisation in past year, diabulimia screening, BMI, method of insulin administration, use of CGM, fingerstick blood glucose testing frequency	Age, gender, ethnicity, marital status, psychiatric diagnoses
Krzyżowska (2023) Poland [78]	Cross-sectional	To evaluate co-occurrence of EDs and depression in adults with T1D and identify implications for nutritional care	HbA_1c_, BMI, diabetes duration, insulin pump treat duration	EAT-26, PHQ-9, CES-D, MDQ, sociodemographic data
Merwin (2014) USA [38]	Cross-sectional	To test whether individuals with T1D are less restrained in their eating when they think their blood glucose is low and whether this contributes to insulin omission for weight management purposes and subsequently higher HbA_1c_	T1D diagnosis, diabetes regimen, history of complications, HbA_1c_, DEPS-R and questionnaire developed by authors measuring disinhibited eating when blood glucose is perceived to be low	Demographic information, eating behaviour
Merwin (2024) USA [7]	Cross-sectional	To identify latent profiles of DEB in adults with T1D using DEPS-R items and evaluate how profiles differ on diabetes-related and psychological outcomes	DEPS-R, HbA_1c_, BMI, diabetes distress, frequency of hypo/hyperglycaemia, insulin pump use	PHQ-9, GAD-7, gender, ethnicity, age, education level
Priesterroth (2025) Germany [65]	Cross-sectional	To identify distinct subtypes of DEBs in adults with T1D and characterise them according to behavioural patterns, clinical features, psychosocial well-being and diabetes-related complications	DEPS-R, HbA_1c_, DSMQ, hypo/hyperglycaemic events requiring external assistance, insulin therapy type, CGM use, BMI, deliberate induction of hypoglycaemia, HFS-II short form, diabetes duration, PAID	PHQ-8, STAI-T, height, weight, ED diagnosis by healthcare professional, age, gender, education level, employment status, relationship status, household composition
Roberts (2025) USA [50]	Cross-sectional	To examine whether fear of hypoglycaemia predicts the likelihood of DEBs in real-world, naturalistic settings over a 3 day ecological momentary assessment period in adults with T1D and clinically significant DEBs	HbA_1c_, blood glucose data available for a subsample via CGM, HFS-II worry subscale, DEPS-R	EDE 16.0, EMA reports of overeating and binge episodes, age, gender
Stancin (1989) USA [39]	Cross-sectional	To describe the specific binge eating and purging behaviours reported in non-medical settings by a sample of young women with IDDM and to explore the relationships among eating pathology, reported diabetic metabolic management and psychological symptomatology	Diabetes background form, self-assessed metabolic management, diabetes-related hospitalisations, DKA episodes in the past year	Demographic information: ethnicity, education level, bulimia screening form based on DSM-3 diagnostic criteria, EDI, SCL-90-R assessing psychiatric symptoms
Tomás (2025) Portugal [77]	Cross-sectional	To study the presence of EDs in young Portuguese adults and adults with T1D and assess how gender, age group, insulin administration method, carbohydrate counting method and BMI influence ED risk	Method of insulin administration (insulin pump vs pen), carbohydrate counting method, BMI, insulin omission, recognition of the concept of diabulimia	EAT-26, age, gender
Usta Sağlam (2025) Turkey [49]	Cross-sectional	To explore the relationship between DEBs, emotion regulation difficulties, anxiety, depression and glycaemic management (HbA_1c_) in adults with T1D	DEPS-R, HbA_1c_, BMI, microvascular complications: nephropathy, retinopathy and neuropathy, insulin pump therapy status, compliance with diabetes follow-ups	EDE-Q, DERS (emotion regulation difficulties), HADS-A and HADS-D
Watt (2022) Australia [67]	Cross-sectional	To establish the prevalence of DEBs and establish their identifiable factors in adults with T1D attending a large tertiary hospital service	DEPS-R, HbA_1c_, number of years since initial diabetes diagnosis, number of DKA episodes requiring admission, BMI	Age, sex, number of hospital admissions in last 12 months, previous diagnoses of an ED
Wisting (2018) Norway [56]	Cross-sectional	To assess the prevalence of DEBs and symptoms of depression and anxiety among adult men and women with T1D, investigate differences between individuals scoring below and above the cut-off for psychopathology and examine patterns of ED psychopathology by age and weight	DEPS-R, NOKLUS measures including HbA_1c_, T1D onset and treatment mode	HADS, clinical data via NOKLUS
Wisting (2020) Norway [57]	Cross-sectional	To investigate the impact of ED psychopathology, illness perceptions, insulin beliefs and coping strategies on metabolic management in adults with T1D, with a specific focus on gender differences	DEPS-R, HbA_1c_, treatment mode, T1D onset	BIPQ, COPE Inventory, BMQ, clinical data via NOKLUS
Wisting (2021) Norway [58]	Cross-sectional	To investigate the impact of psychological aspects (i.e. illness perceptions, coping strategies, insulin beliefs, depression and anxiety), age and BMI, on ED psychopathology among adults with T1D, with a particular focus on gender differences	DEPS-R, HbA_1c_, BMI, diabetes onset, insulin delivery method, number of injections with insulin	BIPQ, BMQ, COPE Inventory, HADS, clinical data via NOKLUS
Colton (2015) Canada [40]	Cohort	To characterise the clinical presentation of individuals with T1D seeking treatment at an academic ED treatment centre and assess the relative effectiveness of an intensive, CBT-based day hospital treatment programme for individuals with T1D	HbA_1c_ scores, DKA, SH, retinopathy, neuropathy, nephropathy, CVD, BMI	EDE-Q using DSM-4 criteria, diagnostic criteria for EDNOS, frequency of specific ED behavioural symptoms, day hospital treatment participation and completion, length of treatment, weight restoration of BMI>20, reductions in ED symptoms/behaviour
Dickens (2015) USA [74]	Cohort	To examine the impact of a multidisciplinary residential treatment programme for women with T1DE on physical and psychological outcomes and the impact of residential treatment duration on outcomes	HbA_1c_ and fructosamine (average blood glucose level over 3 weeks), diabetes duration, BMI	EDI-3, age, ethnicity, relationship status, ED diagnosis, treatment duration for ED
Doyle (2017) USA [59]	Cohort	To assess the prevalence of DEBs in younger adult men and women (18–28 years) with T1D using DEPS-R and identify any clinical characteristics that are associated with DEBs in this population, which may prompt providers to further evaluate young adults for disordered eating, thus enabling earlier detection and intervention	DEPS-R, diabetes duration, age at diagnosis of diabetes, HbA_1c_, current treatment (pump or injections), BMI	No other outcomes assessed
Erthal (2022) Brazil [76]	Cohort	To assess participants’ perceptions about changes in lifestyle and eating and sleeping patterns after 18 months of the COVID-19 pandemic and identify if aspects related to the pandemic (social distancing, COVID-19 infection, behavioural changes and financial difficulties) are predictors of worsening of eating and sleeping parameters	Glycaemic management (self-reported score), presence of diabetes complications (retinopathy, diabetic kidney disease, neuropathy, macrovascular complications such as acute myocardial infarction, stroke and peripheral arterial disease), use of glucose-lowering agents	Age, ethnicity, last weight measurement recorded in medical records, EAT-26, MSQ, perceived changes in eating habits during the pandemic, perceived physical activity habits, ethnicity, social distancing, COVID-19 infection, perception of weight change during the pandemic, other negative impacts during pandemic
Goebel-Fabbri (2008) USA [10]	Cohort	To determine whether insulin restriction increases morbidity and mortality in women with T1D	BMI, PAID, HFS worry subscale, HbA_1c_, diabetes duration, insulin restriction status, presence of medical complications	Age, SCI-R, BSI for psychiatric symptoms, BULIT-R, Eating Disorders Inventory
Goebel-Fabbri (2011) USA [41]	Cohort	To determine the distinguishing characteristics of women who report stopping insulin restriction at 11 years’ follow-up from those continuing to endorse insulin restriction and to distinguish characteristics in participants who continue to use insulin appropriately from new insulin restrictors	BMI; PAID; HFS worry subscale; diabetes duration; diabetes complications; HbA_1c_; insulin restriction status; self-reported diabetes-specific eating and weight concerns, attitudes towards diabetes treatment and its relationship to weight, problems with diabetes self-management	SCI-R; BSI for psychiatric symptoms; BULIT-R; author-created questionnaire that asked participants to rate how much their weight or body shape influenced how they felt about themselves as people
Hirvelä (2025) Finland [86]	Cohort	To assess the incidence of hospital-treated EDs among individuals with T1D vs diabetes-free control individuals and to assess differences in ED treatment (psychotropic medication, outpatient/inpatient care)	Diabetes status, hospital treatment for any cause including diabetes-related complications	Incidence of EDs, new psychotropic medication prescriptions, outpatient and inpatient hospital treatment for EDs and other mental disorders, hospital use for any cause, age, gender, SES, place of residence
Ismail (2024) UK [9]	Cohort	To evaluate whether a new integrated diabetes–psychiatry care model improves biomedical and psychological outcomes in adults with severe T1DE	HbA_1c_, BMI, DKA admissions, SH episodes, CGM/diabetes device use, DEPS-R, DDS-17, composite T1DE risk score, extended 5 year DKA trend, diabetes duration	PHQ-9, GAD-7, number of ED inpatient admissions, number of Mental Health Act detentions, age, gender, ethnicity
Kim (2024) Republic of Korea [85]	Cohort	To determine the risk of incident mental disorders, including EDs, in adults with new-onset T1D compared with the general population without diabetes	BMI, total cholesterol, aspartate aminotransferase, alanine aminotransferase, dyslipidaemia	Mental disorder diagnosis, age, sex, weight, height, systolic and diastolic BP, smoking status, physical activity, income level, comorbidities
Takii (2003) Japan [84]	Cohort	To describe an ‘integrated inpatient therapy’ for individuals with T1D with recurrent binge eating and assess the effectiveness of ‘integrated inpatient therapy’ for women with bulimia nervosa	HbA_1c,_ BMI	Height and weight, psychological and behavioural traits common to EDs, EDI, SDS and STAI
Takii (2008) Japan [83]	Cohort	To investigate which features of EDs, especially the duration of each problematic behaviour related to EDs, are associated with the onset/progression of retinopathy and nephropathy in women with T1D and clinical EDs	HbA_1c_, retinopathy, nephropathy, age at T1D onset, diabetes duration, duration of severe insulin omission, BMI	Age, duration of binge eating, duration of self-induced vomiting, duration of laxative abuse
Cooper (2017) Australia [42]	Case–control	To determine the incidence of and risk factors for psychiatric disorders in early adulthood in individuals with childhood-onset T1D	Age at T1D diagnosis, HbA_1c_, history of SH episodes, number of clinical visits during paediatric management	Anxiety disorders, EDs, mood disorders, personality and behaviour disorders, schizophrenia and psychosis disorders, substance dependence disorders, SES, comorbidities, service use
Cosentino (2023) Italy [43]	Case–control	To investigate the prevalence of orthorexia nervosa in individuals with T1D vs control individuals	HbA_1c_, time in range, time below range CV and use of glycaemic sensor (flash glucose monitor or CGM) retrieved from clinical records	Demographic information from clinical records, orthorexia questionnaires (ORTO-15, DOS), EDE-Q, BSI
Custal (2014) Spain [44]	Case–control	To compare clinical, psychopathological and personality features between two samples of individuals with ED: those with comorbid T1D and those without T1D	HbA_1c_, insulin adherence, insulin misuse	EDI-2 and TCI-R; weight, height, motivational stage of change using a visual analogue scale
Friedman (1998) France [45]	Case–control	For both sexes, to compare the frequency of EDs between individuals with T1D, including a subsample of women considered to be at risk for these disorders and control participants; to specify the type of EDs displayed by individuals with diabetes, according to DSM-3-R criteria; and to examine their relationship to glycaemic management (HbA_1c_ levels and hypoglycaemia) and somatic complications	BMI, mean value of two last HbA_1c_ measurements, number of mild hypoglycaemic episodes in the past week, number of SH episodes in the past year, frequency and type of somatic complications, average insulin intake, self-assessed compliance, mean number of capillary blood glucose measurements per week	Sociodemographic data, EAT, BITE, clinician diagnostic evaluation using LENTCA
Keane (2018) Ireland [71]	Case–control	To compare the prevalence of DEBs in young adults T2D vs non-diabetic control participants	Most recent HbA_1c_, insulin type, insulin mode of administration, BMI	EDE-Q
Shah (2018) USA [87]	Case–control	To examine the gender differences in glycaemic, BP and lipid management, use of advanced diabetes management technologies and occurrence of acute diabetes-related complications in adults with T1D	Most recent HbA_1c_ measurement, diabetes duration, statin use, lipid profiles, frequency of clinic visits, method of insulin delivery, use of CGM, frequency of SMBG, DKA and SH within past 12 months, frequency of missed insulin doses, dyslipidaemia, BMI	Demographics (age, gender, ethnicity), SES (insurance status, household income, education level), smoking status, previous diagnosis of ED, BP and exercise frequency
Ward (1995) UK [28]	Case–control	To compare EDs in people with and without T1D from the same clinic	Diabetes type, age at T1D onset, misuse of insulin, diabetes-related complications, BMI	EDs: diagnosis, age at onset, duration, amenorrhoea, ED weight management methods, treatment history; family history of psychiatric illness; adverse childhood experiences
Balfe (2013) Ireland [29]	Qualitative	To examine the weight loss concerns of young adults with T1D	Barriers to diabetes management in the young adult period	Weight loss activities and ED behaviours
Embick (2025) UK [82]	Qualitative	To develop a recovery model for individuals living with T1DE	Duration of diabetes	Age, gender
Hage (2023) Norway [66]	Qualitative	To investigate the feasibility, using qualitative methodology, of the adapted Body Project for individuals with T1D	Age at T1D onset, DEPS-R	Age, gender
Harrison (2021) UK [46]	Qualitative	To develop a CBT model outlining the development and maintenance of disordered eating in T1D and report on recovery strategies and resilience factors	HbA_1c_, BMI, DDS-17, DEPS-R	PHQ-9
Harrison (2024) UK [4]	Qualitative	To develop the first CBT model outlining the development and maintenance of disordered eating in adult men living with T1D to improve on previous theoretical models of T1D and disordered eating and to draw comparisons with women with T1D and disordered eating	HbA_1c_, diabetes duration, episodes of SH in the last 12 months, episodes of DKA in the last 12 months, DEPS-R, BMI	Age, gender, ethnicity, height, weight
Hastings (2016) UK [88]	Qualitative	To explore how different social identities facilitate or hinder recovery in online diabulimia groups	Experiences of the online support group, impression of support services available for individuals with diabulimia, experiences of seeking help and support from family, friends and HCPs	No other outcomes assessed
Macdonald (2018) UK [30]	Qualitative	To explore patients’ and professionals’ views of treatment of those with T1DE	Patients: experiences of T1DE (start, triggers, life), recovery, services and support received. HCPs: experiences of treating people with T1DE, challenges, need for support, suggestions for improvement	No other outcomes assessed
Falcão (2017) Portugal [47]	Mixed methods	To characterise and compare young adults with and without diabetes with regard to BMI, body image dissatisfaction and disordered eating and evaluate possible predictor variables of disordered eating in both groups; and to explore perceptions of young adults with T1D about its consequences for food intake, body image and weight, and to identify the use of insulin omission behaviour as a weight management strategy	Questionnaire about food and body image (includes insulin omission)	Questionnaire on personal experiences with food and body image, EDE-Q, CDRS
Rama Chandran (2021) UK [89]	Mixed methods	To characterise glycaemia in people with T1DE and explore the association of glycaemic changes with emotion and diabetes self-care behaviours	Blinded CGM data. Meal information, emotions and behaviours around a meal in relation to diabetes, insulin doses, glucose measurements in diabetes diary. DEPS-R, DDS-17, CGM and mySugr satisfaction	Self-reported details about each meal, emotions and behaviours around a meal, exercise; PHQ-9 and YFAS
Verbist (2021) UK [62]	Mixed methods	To examine predictors for the prevalence of DEBs and body image concerns in a T1D population and qualitatively explore links between T1D management and body image concerns	Demographics questionnaire; duration of T1D; DEPS-R; diabetes and body image perception	Demographics questionnaire; weight, height; BISS; social network usage; body image perception
Merwin (2015) USA [60]	EMA	To identify predictors of insulin restriction in the natural environment that might inform new treatment directions	HbA_1c_, CGM, DEPS-R, EMA data on insulin dosing throughout the day, age at T1D diagnosis, duration of diabetes, treatment regimen	BSI, clinician-administered EDE 16.0, EMA data on emotions and eating throughout the day; demographics
Merwin (2018) USA [48]	EMA	To determine the frequency of insulin restriction by time of day	Age at diabetes diagnosis, duration of diabetes, treatment regimen, retinopathy, neuropathy, nephropathy, and DKA, DEPS-R, HbA_1c_, CGM, EMA data on insulin dosing throughout the day, 28 day retrospective report of insulin dosing	Demographic characteristics, EMA data about eating; retrospective eating, clinician-administered adapted EDE
Moskovich (2019) USA [61]	EMA	To examine real-time emotional precursors to and consequences of objective binge eating (OBE) in adults with T1D, and its impact on 2 h postprandial glycaemia vs non-OBE episodes	Age at diabetes diagnosis, duration of diabetes, treatment regimen, EMA data of T1D management behaviour, CGM, HbA_1c_, DEPS-R, hypoglycaemia unawareness	EMA data of mood, eating patterns and demographic characteristics
Broadley (2019) Australia [70]	Quasi-experimental	To explore relationships between food attentional bias scores and disordered eating behaviour in a young adult women with T1D (vs a control group without diabetes) to examine whether unique attentional biases in this group may explain disordered patterns of eating-related thoughts and behaviours	Age at diagnosis, insulin management method, current HbA_1c_ level, experience of any T1D-related complications	Age, gender, height, weight, EDE-Q, attentional bias to food stimuli
Stadler (2025) UK [51]	Feasibility RCT	To test the feasibility, safety and preliminary effectiveness of STEADY, a co-designed CBT-based intervention tailored for adults with T1D and mild-to-moderate disordered eating	HbA_1c_, glucose time in range, CGM wear time, DKA, SH, retinopathy, neuropathy, nephropathy, CVD, autonomic neuropathy, diabetes distress: T1-DDS, DEPS-R, BMI, diabetes duration, HFS-II, DIDP	EDE-QS, PHQ-9, GAD-7, WHOQOL, YBC-EDS-SRQ, BIS/BAS, age, gender, ethnicity, sociodemographic data, SCID-5 RV, mental health diagnoses

BAS, Behaviour Activation System; BDI-II, Beck’s Depression Inventory II; BESAA, Body-Esteem Scale for Adolescents and Adults; BIPQ, Brief Illness Perception Questionnaire; BIS, Behaviour Inhibition System; BISS, Body Image States Scale; BITE, Bulimic Investigatory Test of Edinburgh; BMQ, Beliefs about Medicines Questionnaire; BRIEF-A, Behavior Rating Inventory of Executive Function – Adult version; BSI, Brief Symptom Inventory; BULIT-R, Bulimia Test – Revised; CBT, cognitive behavioural therapy; CD-2, Connor Davidson Resilience Scale; CDRS, Contour Drawing Rating Scale; CES-D, Center for Epidemiological Studies Depression Questionnaire; CGM, continuous glucose monitor; CSII, continuous subcutaneous insulin infusion; DASS-21, Depression, Anxiety and Stress Scale; DDS-17, Diabetes Distress Scale; DEPS-R, Diabetes Eating Problem Survey – Revised; DEB, disordered eating behaviour; DERS, Difficulties in Emotion Regulation Scale; DIDP, DAWN2 Impact of Diabetes Profile; DKA, diabetic ketoacidosis; DOS, Düsseldorf Orthorexie Scale; DSMQ, Diabetes Self-Management Questionnaire; EAT-26, Eating Attitudes 26; ED, eating disorder; EDE-Q, Eating Disorders Examination Questionnaire; EDE-QS, Eating Disorders Examination Questionnaire-Short; EDI, Eating Disorder Inventory; EDI-3RC, Eating Disorder Inventory Risk Composite; EDNOS, Eating Disorder Not Otherwise Specified; EMA, ecological momentary assessment; ERQ, Emotion Regulation Questionnaire; FES, Family Environment Scale; GAD-7, General Anxiety Disorder; HADS, Hospital Anxiety and Depression Scale; HCP, healthcare professional; HFS-II, Hypoglycaemia Fear Survey-II; IDDM, insulin-dependent diabetes mellitus; IPAQ, International Physical Activity Questionnaire; LENTCA, Logistic System for Nosographic Evaluation of Eating Disorders; MDQ, Mood Disorder Questionnaire; m-SCOFF, modified Sick, Control, One stone, Fat, Food; MSPSS, Multidimensional Scale of Perceived Social Support; MSQ, Min-Sleep Questionnaire; NCI FFQ, National Cancer Institute Food Frequency Questionnaire; NOKLUS, Norwegian Quality Improvement of Laboratory Examinations; ORTO-15, Ortorexie-15; PACS-R, Physical Appearance Comparison Scale-Revised; PAID, Problem Areas In Diabetes; PHQ, Patient Health Questionnaire; PSS, Perceived Stress Scale; ROCF-A, Rey–Osterrieth Complex Figure Type A test; SCID-5 RV, Structured Clinical Interview for DSM-5 Research Version; SCI-R, Self-Care Inventory – Revised; SCL-90-R, Symptom Checklist-90 – Revised; SCOFF, Sick, Control, One stone, Fat, and Food; SDS, Zung Self-Rating Depression Scale; SES, socioeconomic status; SH, severe hypoglycaemia; SMBG, self-monitoring of blood glucose; STAI-T, State Trait Anxiety Inventory- Trait version; T1D, type 1 diabetes; T1-DDS, Type 1-Diabetes Distress Scale; T2D, type 2 diabetes; TCI-R, Temperament and Character Inventory – Revised; WHOQOL, World Health Organization Quality Of Life; YBC-EDS-SRQ, Yale-Brown-Cornell Eating Disorders Scale Self-Report Questionnaire; YFAS, Yale Food Addiction Scale

#### Diabetes-related outcomes

**Fig. 3:**
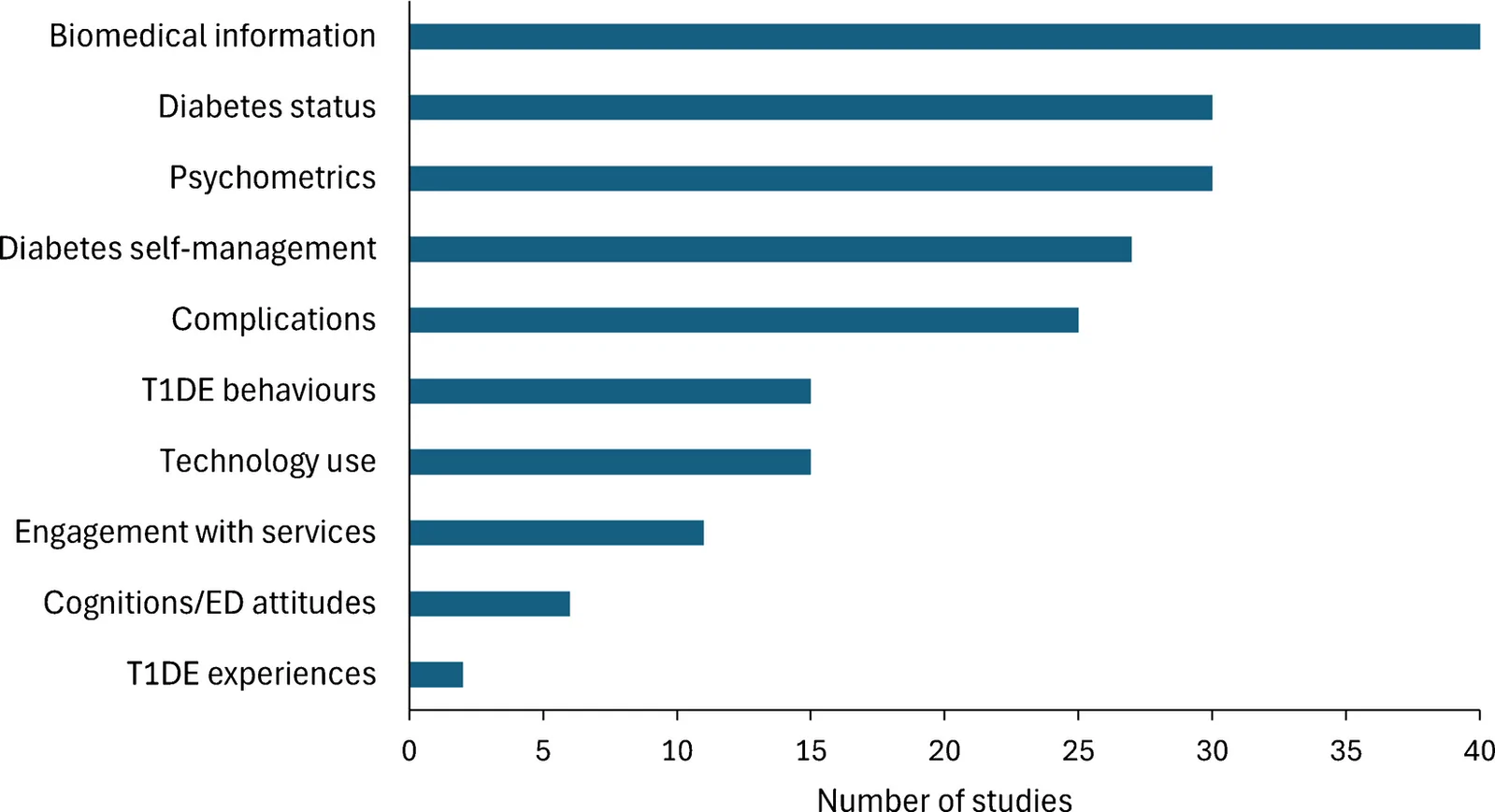
Frequency of reporting of diabetes-related conceptual domains in T1DE across included studies

In total, 201 diabetes-related outcomes were counted across the studies and were grouped into ten conceptual domains (Fig. 3). Each study assessed several different outcomes at a time; hence, the number of outcomes is higher than the number of studies. Most commonly, biomedical information was collected in 40 studies [7, 9, 10, 28, 31, 32, 34, 36–41, 43, 45, 46, 48, 54, 56, 57, 59, 60, 62–67, 70, 71, 74, 75, 77, 78, 80, 81, 83–85, 90]. This included information such as HbA_1c_, BMI, lipid profile and glycaemic variability. Outcomes regarding diabetes status were assessed in 30 studies [4, 9, 10, 28, 29, 32, 37, 41, 42, 47, 48, 50–52, 56, 57, 59–61, 63–68, 74, 78, 81, 83, 87] and included information such as diabetes duration and age at onset. Diabetes-related psychometrics were assessed in 30 studies [4, 7, 9, 10, 35–38, 41, 46, 48–52, 54–57, 59–64, 67, 68, 81, 82, 89] and included the DEPS-R and other questionnaires used to assess diabetes-related distress or quality of life. Diabetes self-management was assessed in 27 studies [31, 32, 34, 36, 38, 39, 44, 45, 48, 49, 52, 54, 56, 57, 59–61, 63, 65, 68, 70, 71, 77, 81, 87, 89, 90] and included information on insulin dosing, blood glucose monitoring, nutrition, exercise, and carbohydrate counting strategies. Diabetes-related complications were assessed in 25 studies and included micro- and macrovascular complications at different levels of severity [4, 7, 9, 10, 28, 31, 38–42, 45, 48, 49, 51, 54, 61, 63, 65, 70, 76, 81, 83, 85, 87]. Data regarding technology use were collected from 15 studies [7, 9, 30, 34, 43, 48, 49, 51, 54, 61, 63–65, 87, 90] and included use of continuous glucose monitors, pumps and diabetes smartphone applications. T1DE behaviours were also assessed in 15 studies [10, 28, 31, 34, 36–38, 41, 44, 52, 65, 77, 83, 87, 89] and included insulin misuse and disinhibited eating triggered by perceived low blood glucose. Engagement with services (i.e. hospitalisations or engagement with support groups) was assessed in 11 studies [30, 31, 36, 39, 42, 67, 81, 86–88, 90]. Participants’ cognitions were assessed in six studies [28, 33, 41, 62, 77, 89] and included overall attitudes toward diabetes in relation to weight. Finally, T1DE experiences were explored in two studies [4, 30] and included experiences relating to living with T1DE (onset, maintenance) and recovery factors.

#### Other outcomes

**Fig. 4:**
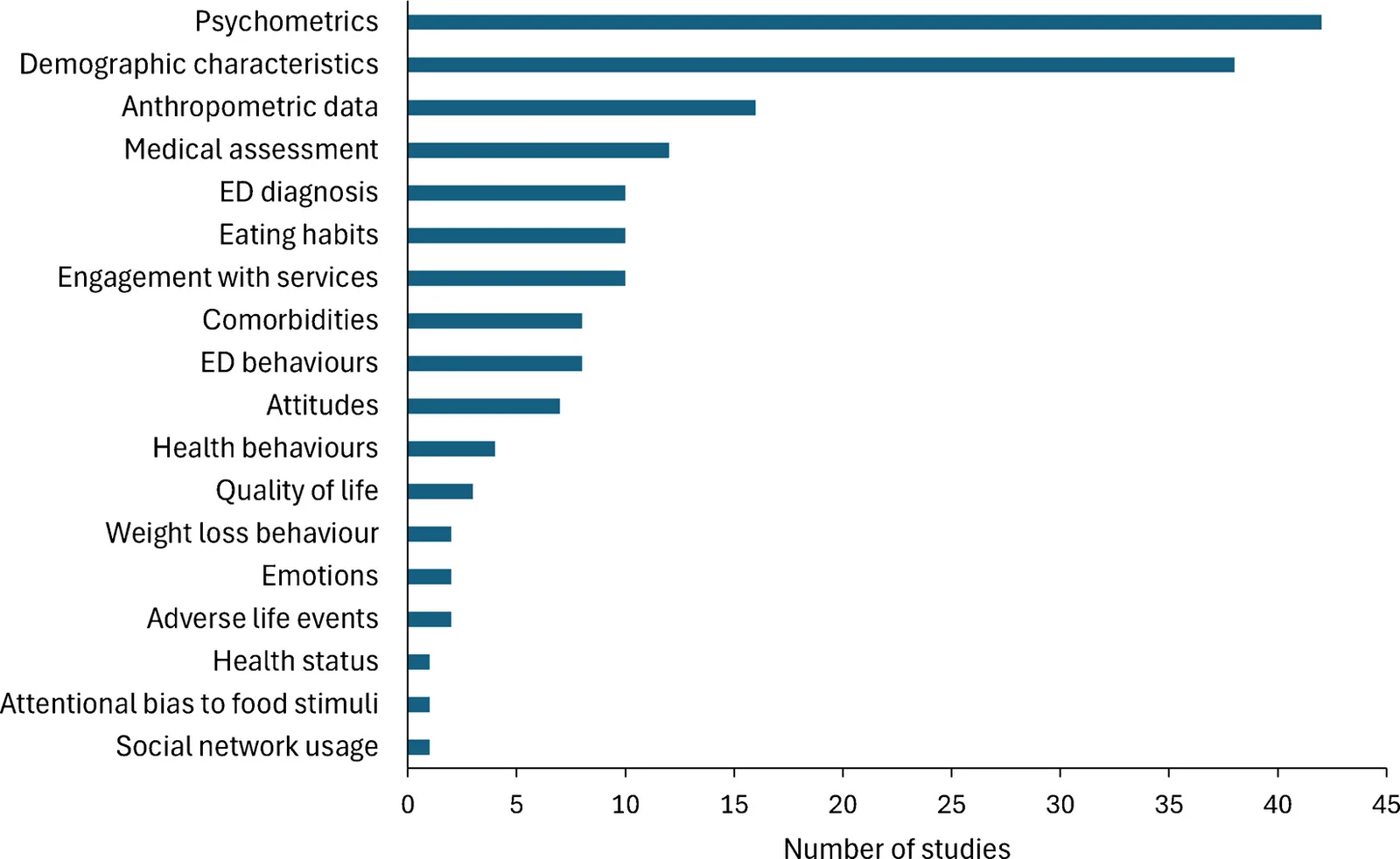
Frequency of reporting of eating disorder (ED)-related conceptual domains in T1DE across included studies

The included studies assessed another 177 outcomes, which were categorised into 18 conceptual domains (Fig. 4). Psychometric tests were assessed in 42 studies [7, 9, 10, 31–37, 39–41, 43–52, 54–57, 60, 62, 65, 68, 70, 71, 74–78, 80, 81, 84, 89] and covered eating disorders and other psychopathology, quality of life, cognition, social support, resilience, psychological distress, financial concerns, emotional regulation, appearance perceptions, illness perception, health status and self-care. Demographic characteristics were assessed in 38 studies [4, 7, 9, 10, 31, 37–39, 42, 43, 45, 48, 50–52, 54, 60–67, 70, 74–78, 80–83, 85–87, 90]. Anthropometric data (e.g. BMI) were collected in 16 studies [4, 32–34, 44, 54, 62, 63, 65, 70, 75, 76, 81, 84, 85, 87]. Medical assessment was performed in 12 studies [32–34, 40, 45, 54, 56–58, 75, 81, 86] and included biochemical assessments, medical history and medication intake. Engagement with eating disorder-related services [9, 28, 40, 42, 54, 64, 67, 74, 81, 86], eating habits [31, 32, 34, 38, 48, 54, 60, 61, 76, 89] and eating disorder diagnosis [28, 40, 42, 52, 65, 67, 74, 81, 86, 87] were each assessed in ten studies. Eating disorder behaviours, such as binge eating, vomiting and laxative use, were assessed in eight studies [28, 29, 40, 50, 54, 83, 84, 89]. Psychological or psychiatric comorbidities [28, 42, 51, 76, 81, 85, 90], eating disorder attitudes [33, 41, 44, 52, 54, 62, 76], and included cognitions related to body image, eating and weight were assessed in seven studies. Information on health behaviours was collected in four studies [31, 54, 76, 87] and included exercise and social distancing during the COVID-19 pandemic. Quality of life was assessed in three studies [34, 64, 85], and emotions that were not directly linked to diabetes [60, 61], adverse life events [28, 76] and weight loss behaviours [29, 54] such as being on a diet were each assessed in two studies. Finally, attentional bias to food stimuli [70], social network usage [62] and self-reported health status [52] were each assessed in one study.

## Discussion

This review identified substantial heterogeneity in T1DE definitions. Across 61 studies, 29 different questionnaires were used, with the DEPS-R being the most common. Similar heterogeneity has been noted previously [13, 91] and contributes to inconsistent prevalence estimates, diagnostic criteria and interpretation of findings. The included studies examined diabetes-related outcomes (*n*=201) and eating disorder-related outcomes (*n*=177), reflecting a fragmented understanding that separates diabetes and disordered eating rather than integrating them. Clinical assessments allowed for a systematic evaluation of eating disorders, but the lack of T1DE-specific criteria meant that links between diabetes care and disordered eating had to be inferred, complicating the interpretation of findings and hindering the development of effective screening tools and clear care pathways.

Recent work [46, 92] and updated guidance [16] indicate a shift toward conceptualising T1DE as a multifaceted condition requiring interdisciplinary care. Nevertheless, current definitions remain predominantly grounded in medical models, emphasising biomedical markers over psychological components.

Without a formal, universally accepted definition, individuals may be classified under Eating Disorder Not Otherwise Specified (EDNOS)/Other Specified Feeding or Eating Disorder (OSFED) or ‘diabulimia’, terms that fail to capture the complexity of T1DE and patient care needs [8, 93]. Emerging T1DE subtype frameworks exist [7], but current diagnostic manuals offer inadequate guidance, focusing primarily on insulin omission [94–96]. Consequently, the synthesised literature reflects this focus on insulin omission and does not fully represent all T1DE ‘phenotypes’.

This is the first review to systematically map the diverse definitions and assessment methods for T1DE, providing a comprehensive overview. By integrating a wide array of definitions and questionnaires, we highlight considerable variability in how T1DE is conceptualised, offering a starting point for the development of universal consensus criteria. The overall quality of the included studies was good, particularly regarding internal validity.

However, the integration of heterogeneous definitions was a limitation. For example, we could not synthesise prevalence estimates for T1DE or insulin omission, due to inconsistent reporting across studies. This remains an important area of research, particularly when integrated with objective glycaemic data. Additionally, language restrictions (English) may have introduced bias and limited global applicability. The focus on adults limits applicability to adolescents, and men were also under-represented, reflective of the general eating disorders literature. T1DE may present clinically differently in men [4], or be more hidden due to societal/cultural factors. Most studies were conducted in high-income countries, limiting generalisability. External validity was generally weaker than internal validity, and interdisciplinary expertise in both type 1 diabetes and eating disorders was limited. Some integrative factors (i.e. excessive exercise) were underexplored. Finally, potential overlap in participant samples could not be fully assessed, although no quantitative synthesis was conducted, so this did not affect the interpretation of findings.

This review focused on definitions and assessment criteria, unlike previous reviews that have centred on interventions [92] or prevalence estimates [91]. The previous review by Pursey et al [13] focused on validated questionnaires and adolescents, whereas this review adopts a broader perspective by including both validated and non-validated questionnaires and focusing specifically on adults [13]. Hall et al [97] concentrated on risk factors associated with insulin omission, whereas this review also incorporated a wider range of T1DE-related behaviours. Additionally, one study focused on men’s experiences of insulin restriction [98], whereas this review included both genders/sexes and a more diverse participant population.

Two studies published in 2024 warrant mentioning. One is a meta-analysis of T1DE prevalence [91], which fell outside the scope of this review. The other, by Merwin et al [7], used the DEPS-R alongside blood glucose data to develop behavioural profiles of T1DE subtypes. This contrasts with the current review, which examined a broader range of assessment approaches. Together, these findings indicate an urgent need for a consensus-based diagnostic approach, incorporating multifactorial criteria that reflect the psychological, behavioural and biomedical complexity of T1DE.

The absence of a clear, overarching definition of T1DE has significant clinical consequences. A universal T1DE definition would mark the pathway for developing suitable, effective treatments, interdisciplinary collaborations to help differentiate T1DE from other conditions, and appropriate clinician training. Without standard criteria, clinicians may feel ill-prepared, leading to delays in intervention, and patients may cycle through services without adequate support [30, 99].

From a policy perspective, a unified definition would inform the development of clear guidelines to inform resource allocation and shape professional training programmes. It would also encourage the creation of integrated care models, in which multidisciplinary teams work together to provide holistic care, improving physical and mental health outcomes, while also reducing unnecessary healthcare use. Finally, establishing standardised criteria would set the stage for more rigorous research trials and the development of evidence-based interventions tailored to the complexities of T1DE.

A formal diagnostic framework that integrates psychological, behavioural and biomedical features of T1DE is urgently needed. To ensure its relevance and acceptability, it should be co-produced with patients and clinicians. Once it is in place, research should focus on developing and evaluating interventions tailored to T1DE, aiming to improve treatment outcomes. Further research should also consider cultural and socioeconomic contexts, exploring whether the presentation, recognition and management of T1DE vary across different regions and healthcare systems. In routine clinical practice, a standardised definition for T1DE would enable earlier identification and risk stratification, provide a shared clinical language for diabetes and mental health teams, and clarify referral and treatment pathways across services.

### Conclusion

This narrative systematic review highlights the significant heterogeneity in T1DE definition and screening, often reflecting a medical model that may undervalue psychological and behavioural aspects. Addressing this requires a holistic, evidence-based diagnostic framework that operationalises insulin misuse and intent, distinguishes clinical presentations and is applicable across gender/sex and cultural contexts. This will improve accurate identification, intervention development, and person-centred care.

## Supplementary Information

Below is the link to the electronic supplementary material.

ESM Tables (PDF 227 KB)

## Data Availability

As this is a secondary analysis of existing literature, all raw data were obtained from published reports and are cited in accordance with ICMJE guidelines. Analysed data, including individual study characteristics and outcome estimates, are provided in the electronic supplementary material. The complete data extraction form and study protocol are available from the corresponding author on reasonable request beginning 3 months and ending 5 years following article publication.

## Abbreviations

DEPS-R: Diabetes Eating Problem Survey – Revised
DSM: Diagnostic and Statistical Manual of Mental Disorders
EDE-Q: Eating Disorder Examination – Questionnaire
EDI: Eating Disorders Inventory
EMA: Ecological Momentary Assessment
SCOFF: Sick, Control, One stone, Fat, and Food
T1DE: Type 1 diabetes and disordered eating
