# Supplementary material for: A narrative systematic review of definitions and diagnostic criteria for disordered eating and eating disorders in type 1 diabetes
Source: Diabetologia. 2026 Jul 29;69(10):2712–40. doi: 10.1007/s00125-026-06805-3 (PMC13541810; doi:10.1007/s00125-026-06805-3)
Supplement: Supplementary file 1 [file 125_2026_6805_MOESM1_ESM.pdf]

Electronic Supplementary Material (ESM)

ESM Table 1: PRISMA statement

Section and Topic	Item #	Checklist item	Location where item is reported
**TITLE**			
Title	1	Identify the report as a systematic review.	Pg 1
**ABSTRACT**			
Abstract	2	See the PRISMA 2020 for Abstracts checklist.	Pgs 2-3
**INTRODUCTION**			
Rationale	3	Describe the rationale for the review in the context of existing knowledge.	Pgs 7-8
Objectives	4	Provide an explicit statement of the objective(s) or question(s) the review addresses.	Pg 8
**METHODS**			
Eligibility criteria	5	Specify the inclusion and exclusion criteria for the review and how studies were grouped for the syntheses.	Pg 8
Information sources	6	Specify all databases, registers, websites, organisations, reference lists and other sources searched or consulted to identify studies. Specify the date when each source was last searched or consulted.	Pgs 8-9
Search strategy	7	Present the full search strategies for all databases, registers and websites, including any filters and limits used.	Pg 9 &ESM Table 2
Selection process	8	Specify the methods used to decide whether a study met the inclusion criteria of the review, including how many reviewers screened each record and each report retrieved, whether they worked independently, and if applicable, details of automation tools used in the process.	Pg 9
Data collection process	9	Specify the methods used to collect data from reports, including how many reviewers collected data from each report, whether they worked independently, any processes for obtaining or confirming data from study investigators, and if applicable, details of automation tools used in the process.	Pg 9

Data items	10a	List and define all outcomes for which data were sought. Specify whether all results that were compatible with each outcome domain in each study were sought (e.g. for all measures, time points, analyses), and if not, the methods used to decide which results to collect.	Pg 9
	10b	List and define all other variables for which data were sought (e.g. participant and intervention characteristics, funding sources). Describe any assumptions made about any missing or unclear information.	Pg 9
Study risk of bias assessment	11	Specify the methods used to assess risk of bias in the included studies, including details of the tool(s) used, how many reviewers assessed each study and whether they worked independently, and if applicable, details of automation tools used in the process.	Pg 9
Effect measures	12	Specify for each outcome the effect measure(s) (e.g. risk ratio, mean difference) used in the synthesis or presentation of results.	N/A
Synthesis methods	13a	Describe the processes used to decide which studies were eligible for each synthesis (e.g. tabulating the study intervention characteristics and comparing against the planned groups for each synthesis (item #5)).	Pg 9
	13b	Describe any methods required to prepare the data for presentation or synthesis, such as handling of missing summary statistics, or data conversions.	Pg 10
	13c	Describe any methods used to tabulate or visually display results of individual studies and syntheses.	Pg 10
	13d	Describe any methods used to synthesize results and provide a rationale for the choice(s). If meta-analysis was performed, describe the model(s), method(s) to identify the presence and extent of statistical heterogeneity, and software package(s) used.	Pg 10
	13e	Describe any methods used to explore possible causes of heterogeneity among study results (e.g. subgroup analysis, meta-regression).	N/A

	13f	Describe any sensitivity analyses conducted to assess robustness of the synthesized results.	N/A
Reporting bias assessment	14	Describe any methods used to assess risk of bias due to missing results in a synthesis (arising from reporting biases).	N/A
Certainty assessment	15	Describe any methods used to assess certainty (or confidence) in the body of evidence for an outcome.	N/A
**RESULTS**			
Study selection	16a	Describe the results of the search and selection process, from the number of records identified in the search to the number of studies included in the review, ideally using a flow diagram.	Pgs 10
	16b	Cite studies that might appear to meet the inclusion criteria, but which were excluded, and explain why they were excluded.	NR
Study characteristics	17	Cite each included study and present its characteristics.	Table 2
Risk of bias in studies	18	Present assessments of risk of bias for each included study.	ESM Table 3
Results of individual studies	19	For all outcomes, present, for each study: (a) summary statistics for each group (where appropriate) and (b) an effect estimate and its precision (e.g. confidence/credible interval), ideally using structured tables or plots.	N/A
Results of syntheses	20a	For each synthesis, briefly summarise the characteristics and risk of bias among contributing studies.	Table 2 and ESM Table 3
	20b	Present results of all statistical syntheses conducted. If meta-analysis was done, present for each the summary estimate and its precision (e.g. confidence/credible interval) and measures of statistical heterogeneity. If comparing groups, describe the direction of the effect.	N/A
	20c	Present results of all investigations of possible causes of heterogeneity among study results.	N/A
	20d	Present results of all sensitivity analyses conducted to assess the robustness of the synthesized results.	N/A

Reporting biases	21	Present assessments of risk of bias due to missing results (arising from reporting biases) for each synthesis assessed.	N/A
Certainty of evidence	22	Present assessments of certainty (or confidence) in the body of evidence for each outcome assessed.	N/A
**DISCUSSION**			
Discussion	23a	Provide a general interpretation of the results in the context of other evidence.	Pgs 18-21
	23b	Discuss any limitations of the evidence included in the review.	Pgs 19-20
	23c	Discuss any limitations of the review processes used.	Pgs 19-20
	23d	Discuss implications of the results for practice, policy, and future research.	Pgs 20-21
**OTHER INFORMATION**			
Registration and protocol	24a	Provide registration information for the review, including register name and registration number, or state that the review was not registered.	Pg 8
	24b	Indicate where the review protocol can be accessed, or state that a protocol was not prepared.	Pg 8
	24c	Describe and explain any amendments to information provided at registration or in the protocol.	N/A
Support	25	Describe sources of financial or non-financial support for the review, and the role of the funders or sponsors in the review.	The systematic review was funded as part of the PhD Studentship – JMRC-KRT
Competing interests	26	Declare any competing interests of review authors.	N/A
Availability of data, code and other materials	27	Report which of the following are publicly available and where they can be found: template data collection forms; data extracted from included studies; data used for all analyses; analytic code; any other materials used in the review.	No data is available publicly, but can be accessed upon contacting the corresponding author

From: Page MJ, McKenzie JE, Bossuyt PM, Boutron I, Hoffmann TC, Mulrow CD, et al. The PRISMA 2020 statement: an updated guideline for reporting systematic reviews. BMJ 2021;372:n71. doi: 10.1136/bmj.n71

ESM Table 2: Search Strings

MEDLINE example search string

**Database: MEDLINE**<br>**Date of search: 20/06/2023**<br>**Limits: December 2022-Present**<br>**Layer 1 AND Layer 2. Hits: 2495**		
**Layer 1**	1	exp Diabetes Mellitus, Type 1/
	2	(DKA or IDDM).mp. or DMI.ab,ti.
	3	insulin* secret* dysfunc*.ti,ab.
	4	insulin* depend*.mp. or insulin?depend*
	5	(("typ* 1" or typ* I) adj6 DM).ti,ab.
	6	((juvenil$ or child$ or keto$ or labil$ or brittl$ or earl$ onset) adj6 (DM or DM1 or diabetes)).ti,ab.
	7	((keto$ prone or autoimmun$ or auto immun$ or sudden onset) adj6 (DM or DM1)).ti,ab.
	8	1-7 (OR)
**AND**		
**Layer 2**	9	exp "Feeding and Eating Disorders"/
	10	diabulimia.mp
	11	disordered eating.mp.
	12	Body Dysmorphic Disorders/
	13	body image/ or body dissatisfaction/
	14	muscle dysmorphia.mp.
	15	insulin omission.mp.
	16	insulin restriction.mp.
	17	Anorexi*.ti,ab,kw,ot.
	18	(eat* adj3 disorder).ti,ab,kw,ot.
	19	Bulimia/
	20	Bulimi*.ti,ab,ot,kw.

	21	EDNOS.ti,ab,ot,kw.
	22	(binge adj3 eat$).ti,ab,kw,ot.
	23	diet therapy/ or caloric restriction/ or diet, carbohydrate loading/ or diet, diabetic/ or diet, carbohydrate-restricted/ or diet, high-protein low-carbohydrate/ or diet, ketogenic/
	24	Carbohydrate restriction.mp.
	25	Androgens/
	26	Anabolic Agents/
	27	Performance-Enhancing Substances/
	28	Steroids/
	29	anabolic androgenic steroids.mp.
	30	(recurrent adj3 DKA).mp.
	31	9-30 (OR)

PubMed example search string

**Database: PubMed**		
**Date of search: 20/06/2023**		
**Limits: 2020-Present**		
**Layer 1 AND Layer 2. Hits: 140**		
**Layer 1**	1	"diabetes mellitus, type 1"[MeSH Terms] OR "type 1 diabetes mellitus"[All Fields] OR "type 1 diabetes"[All Fields]
**AND**		
**Layer 2**	2	feeding and eating disorders[MeSH Terms]
	3	diabulimia[All Fields]
	4	disordered eating[All Fields]
	5	body dysmorphic disorders[MeSH Terms] OR "body dysmorphic disorders"[All Fields]
	6	"body image"[All Fields] OR body dissatisfaction[MeSH Terms]
	7	muscle dysmorphia[All Fields]
	8	insulin omission[All Fields] OR "insulin omission"[All Fields]
	9	EDNOS[All Fields]

	10	caloric restriction[MeSH Terms]
	11	carbohydrate restriction[All Fields]
	12	Performance-Enhancing Substances[Mesh]
	13	Withholding[All Fields] AND ("insulin"[MeSH Terms] OR "insulin"[All Fields])

PsycINFO example search string

**Database: PsycINFO**<br>**Date of search: 20/06/2023**<br>**Limits: None**<br>**Layer 1 AND Layer 2. Hits: 409**		
**Layer 1**	1	exp diabetes mellitus/
	2	type 1 diabetes.mp.
	3	insulin* depend*.mp. or insulin?depend*
	4	(("typ* 1" or typ* I) adj6 DM).ti,ab.
	5	((juvenil$ or child$ or keto$ or labil$ or brittl$ or earl$ onset) adj6 (DM or DM1 or diabetes)).ti,ab.
	6	((keto$ prone or autoimmun$ or auto immun$ or sudden onset) adj6 (DM or DM1)).ti,ab.
	7	1 or 2 or 3 or 4 or 5 or 6
**AND**		
**Layer 2**	8	exp eating disorders/
	9	diabulimia.mp.
	10	disordered eating.mp.
	11	exp body dysmorphic disorder/
	12	body image/ or body dissatisfaction/
	13	muscle dysmorphia.mp.
	14	exp Anorexia Nervosa/ or exp Bulimia/ or exp Body Image Disturbances/ or anorexia.mp.
	15	insulin omission.mp.
	16	insulin restriction.mp.

	17	exp Binge Eating Disorder/
	18	EDNOS.mp.
	19	exp food refusal/
	20	exp Dietary Restraint/ or exp Food Deprivation/
	21	caloric restriction.mp.
	22	carbohydrate restriction.mp.
	23	androgens/ or exp testosterone/
	24	exp performance enhancing drugs/
	25	insulin mismanagement.mp.
	26	8 or 9 or 10 or 11 or 12 or 13 or 14 or 15 or 16 or 17 or 18 or 19 or 20 or 21 or 22 or 23 or 24 or 25

CINAHL example search string

**Database: CINAHL**<br>**Date of search: 20/06/2023**<br>**Limits: None**<br>**Layer 1 AND Layer 2. Hits: 806**		
**Layer 1**	1	(MM "Diabetes Mellitus, Type 1")
	2	""type 1 diabetes or t1d or diabetes mellitus or juvenile diabetes or insulin-dependent diabetes""
**AND**		
**Layer 2**	7	(MH "Eating Disorders+")
	8	"disordered eating"
	9	"body dissatisfaction"
	10	""body dysmorphic disorder OR bdd OR body dysmorphia"
	11	"caloric restriction"
	12	"muscle dysmorphia"
	13	(MM "Body Dysmorphic Disorder")
	14	""diabulimia or type 1 diabetes and eating disorder or type 1 diabetes and disturbed eating""

	15	"negative body image"
	16	"EDNOS"
	17	"carbohydrate restriction"
	18	"performance enhancing substances"

Embase example search string

**Database: Embase**		
**Date of search: 20/06/2023**		
**Limits: None**		
**Layer 1 AND Layer 2. Hits: 1299**		
**Layer 1**	1	exp insulin dependent diabetes mellitus/
**AND**		
**Layer 2**	2	diabulimia.mp.
	3	exp eating disorder/
	4	EDNOS.mp.
	5	disordered eating.mp.
	6	exp body dysmorphic disorder/
	7	body image/ or body dissatisfaction/
	8	insulin omission.mp.
	9	insulin restriction.mp.
	10	insulin mismanagement.mp.
	11	exp caloric restriction/
	12	carbohydrate restriction.mp.
	13	exp performance enhancing substance/
	14	2 or 3 or 4 or 5 or 6 or 7 or 8 or 9 or 10 or 11 or 12 or 13

ESM Table 3: Population and study characteristics

Author, year, country	Study design	Sample size	Population characteristics	% of women	External validity	Internal validity
Affenito (1997) USA (31)	Cross-sectional	90	T1DE: 27 T1DM: 63	100%	+	+
Affenito (1997) USA (32)	Cross-sectional	90	T1DE: 27 T1DM: 63	100%	+	++
Affenito (1998) USA (33)	Cross-sectional	90	T1DE: 27 T1DM: 63	100%	+	+
Albaladejo (2023) France (34)	Cross-sectional	201	T1DM: 201	31%	++	++
Apergi (2023) Greece (54)	Cross-sectional	100	T1DM: 100	71%	++	++
Babayeva (2025) (63)	Cross-sectional	120	T1DM: 80 T2DM: 40	63%	+	+
Bächle (2015) Germany (80)	Cross-sectional	211	T1DM: 211	60%	++	++
Bikri (2021) Morocco (75)	Cross-sectional	140	T1DM: 140	46%	+	+
Broadley (2018) Australia (35)	Cross-sectional	275	T1DM: 74 No DM: 201	81%	++	++
d'Emden (2017) Australia (36)	Cross-sectional	151	T1DM: 151	56%	++	+
Embaye (2023) The Netherlands (55)	Cross-sectional	291	T1DM: 291	79%	+	+

Ercolino (2025) USA (64)	Cross-sectional	77	T1DM: 77	55%	+	+
Gawlik (2016) Australia (37)	Cross-sectional	177	T1DM: 177	100%	-	++
Haurtlaub (2025) 22 countries, predominantly USA and UK (52)	Cross-sectional	199	T1DM: 199	55%	+	+
Huisman (2023) The Netherlands (81)	Cross-sectional	1317	T1DM: 582<br>T2DM: 735	55%	++	+
Ip (2023) USA (90)	Cross-sectional	225	T1DM: 225	79%	+	+
Krzyżowska, (2023) Poland (78)	Cross-sectional	27	T1DM: 27	56%	+	+
Merwin (2014) USA (38)	Cross-sectional	276	T1DM: 276	69%	+	+
Merwin (2024) USA (7)	Cross-sectional	326	T1DM: 326	72%	+	+
Priesterroth (2025) Germany (65)	Cross-sectional	645	T1DM: 645	71%	+	+
Roberts (2025) USA (50)	Cross-sectional	59	T1DE: 59	93%	+	+
Stancin (1989) USA (39)	Cross-sectional	59	T1DM: 59	100%	+	+
Tomás (2025) Portugal (77)	Cross-sectional	47	T1DM: 47	79%	+	+

Usta Sağlam (2025) Turkey (49)	Cross-sectional	103	T1DM: 103	57%	+	+
Watt (2022) Australia (67)	Cross-sectional	199	T1DM: 199	50%	+	++
Wisting (2018) Norway (56)	Cross-sectional	282	T1DM: 282	60%	+	++
Wisting (2020) Norway (57)	Cross-sectional	282	T1DM: 282	60%	+	+
Wisting (2021) Norway (58)	Cross-sectional	282	T1DM: 282	60%	+	+
Colton (2015) Canada (40)	Cohort	896	T1DE: 95<br>ED: 801	100%	-	+
Dickens (2015) USA (74)	Cohort	29	T1DE: 29	100%	+	+
Doyle (2017) USA (59)	Cohort	60	T1DM: 60	45%	++	++
Erthal (2022) Brazil (76)	Cohort	150	T1DM: 58<br>T2DM: 92	59%	+	++
Goebel-Fabbri (2008) USA (10)	Cohort	234	T1DM: 234	100%	+	++
Goebel-Fabbri (2011) USA (41)	Cohort	207	T1DM: 207	100%	+	++
Hirvelä (2025) Finland (86)	Cohort	22,110	T1DE: 11,055<br>No DM: 11,055	48%	++	++
Ismail (2024) UK (9)	Cohort	66	T1DE: 66	95%	+	+
Kim (2024) Republic of Korea (85)	Cohort	62,386	T1DM: 10.391<br>No DM: 51,995	34%	+	+
Takii (2003)	Cohort	19	T1DE: 19	100%	+	++

Japan (84)						
Takii (2008) Japan (83)	Cohort	109	T1DE: 109	100%	+	++
Cooper (2017) Australia (42)	Case-control	7724	T1DM: 1302<br>No DM: 6422	50%	++	++
Cosentino (2023) Italy (43)	Case-control	88	T1DM: 44<br>No DM: 44	61%	++	++
Custal (2014) Spain (44)	Case-control	40	T1DE: 20<br>ED: 20	100%	++	++
Friedman (1998) France (45)	Case-control	168	T1DM: 69<br>Medical outpatients: 45<br>HCPs: 54	65%	-	-
Keane (2018) Ireland (71)	Case-control	274	T1DM: 49<br>No DM: 225	77%	++	++
Shah (2018) USA (87)	Case-control	9481	T1DM: 9481	53%	++	++
Ward (1995) UK (28)	Case-control	34	T1DE: 17<br>T1DM: 17	NR	-	-
Balfe (2013) Ireland (29)	Qualitative	48	T1DM: 35<br>HCPs: 13	88%	+	
Embick (2025) UK (82)	Qualitative	13	T1DE: 13	85%	++	
Hage (2023) Norway (66)	Qualitative	17	T1DE: 17	100%	++	
Harrison (2021) UK (46)	Qualitative	23	T1DE: 14<br>T1DM: 9	100%	++	
Harrison (2024) UK (4)	Qualitative	27	T1DE: 16<br>T1DM: 11	0%	++	
Hastings (2016) UK (88)	Qualitative	13	T1DE: 13	100%	++	

Macdonald (2018) UK (30)	Qualitative	17	T1DE: 9 HCPs: 8	100%	++	
Rama Chandran (2021) UK (89)	Mixed methods	23	T1DE: 13 T1DM: 10	100%	+	++
Falcao (2017) Portugal (47)	Mixed methods	128	T1DM: 55 No DM: 73	77%	+	++
Verbist (2021) UK (62)	Mixed methods	121	T1DM: 121	88%	+	+
Merwin (2015) USA (60)	Ecological Momentary Assessment	83	T1DE: 83	88%	++	++
Merwin (2018) USA (48)	Ecological Momentary Assessment	59	T1DE: 59	93%	++	++
Moskovich (2019) USA (61)	Ecological Momentary Assessment	83	T1DE: 83	88%	++	++
Broadley (2019) Australia (70)	Quasi-experimental	97	T1DM: 41 No DM: 56	100%	++	++
Stadler (2025) UK (51)	Feasibility Randomised Controlled Trial (RCT)	40	T1DE: 40	95%	++	+

T1DE: Type 1 diabetes and disordered eating, T1DM: Type 1 diabetes mellitus, T2DM: Type 2 diabetes mellitus, No DM: No diagnosis of diabetes, ED: Diagnosis of an eating disorder only, HCPs: Healthcare professionals.

++ : All or most criteria have been fulfilled (low risk of bias)

+ : Some criteria have been fulfilled (unclear risk of bias)

- : Few or no criteria have been fulfilled (high risk of bias)
